# Nutritional and health status of children 15 months after integrated school garden, nutrition, and water, sanitation and hygiene interventions: a cluster-randomised controlled trial in Nepal

**DOI:** 10.1186/s12889-019-8027-z

**Published:** 2020-02-03

**Authors:** Akina Shrestha, Christian Schindler, Peter Odermatt, Jana Gerold, Séverine Erismann, Subodh Sharma, Rajendra Koju, Jürg Utzinger, Guéladio Cissé

**Affiliations:** 1 Swiss Tropical and Public Health Institute, P.O. Box, CH-4002, Basel, Switzerland; 2 University of Basel, P.O. Box, CH-4003, Basel, Switzerland; 3 School of Medical Sciences, Kathmandu University, Dhulikhel, Nepal; 4 School of Science, Aquatic Ecology Centre, Kathmandu University, Dhulikhel, Nepal

**Keywords:** Anaemia, Intestinal parasitic infections, Malnutrition, Nepal, School-aged children, School garden, Water, sanitation and hygiene (WASH)

## Abstract

**Background:**

It has been suggested that specific interventions delivered through the education sector in low- and middle-income countries might improve children’s health and wellbeing. This cluster-randomised controlled trial aimed to evaluate the effects of a school garden programme and complementary nutrition, and water, sanitation and hygiene (WASH) interventions on children’s health and nutritional status in two districts of Nepal.

**Methods:**

The trial included 682 children aged 8–17 years from 12 schools. The schools were randomly allocated to one of three interventions: (a) school garden programme (SG; 4 schools, *n* = 172 children); (b) school garden programme with complementary WASH, health and nutrition interventions (SG+; 4 schools, *n* = 197 children); and (c) no specific intervention (control; 4 schools, *n* = 313 children). The same field and laboratory procedures were employed at the baseline (March 2015) and end-line (June 2016) surveys. Questionnaires were administered to evaluate WASH conditions at schools and households. Water quality was assessed using a Delagua kit. Dietary intake was determined using food frequency and 24-h recall questionnaire. Haemoglobin levels were measured using HemoCue digital device and used as a proxy for anaemia. Stool samples were subjected to a suite of copro-microscopic diagnostic methods for detection of intestinal protozoa and helminths. The changes in key indicators between the baseline and end-line surveys were analysed by mixed logistic and linear regression models.

**Results:**

Stunting was slightly lowered in SG+ (19.9 to 18.3%; *p* = 0.92) and in the control (19.7 to 18.9%). Anaemia slightly decreased in SG+ (33.0 to 32.0%; *p* < 0.01) and markedly increased in the control (22.7 to 41.3%; *p* < 0.01), a minor decline was found in the control (43.9 to 42.4%). Handwashing with soap before eating strongly increased in SG+ (from 74.1 to 96.9%; *p =* 0.01, compared to control where only a slight increase was observed from 78.0 to 84.0%). A similar observation was made for handwashing after defecation (increase from 77.2 to 99.0% in SG+ versus 78.0 to 91.9% in control, *p* = 0.15).

**Conclusions:**

An integrated intervention consisting of school garden, WASH, nutrition and health components (SG+) increased children’s fruit and vegetable consumption, decreased intestinal parasitic infections and improved hygiene behaviours.

**Trial registration:**

ISRCTN17968589 (date assigned: 17 July 2015).

## Background

Childhood is a critical period for the development of eating patterns that persist into adulthood, particularly with regards to fruit and vegetable consumption [1]. Hence, it is vital that children learn early about the importance of a balanced diet, including fruits and vegetables [2]. Considering the importance of adequate nutrition in childhood to achieve healthy growth and development, giving children opportunities to learn about fruits and vegetables, including their benefits, may help to facilitate the increase in their intake that could prevent malnutrition [1]. School gardens are considered as an ideal setting to facilitate dietary behaviour change among children. They offer a potential to increase children’s exposure to, and consumption of, fruits and vegetables [3]. Studies indicate positive effects on children’s food preferences and dietary habits, including fruits and vegetables consumption, and about knowledge, benefit towards good health and prevention of malnutrition [4, 5]. School garden education also provides a context for understanding seasonality, what needs to be eaten and where food comes from [1, 6]. Furthermore, it provides an opportunity to teach life skills to school-aged children, including gardening and working cooperatively on planting and harvesting [1].

Malnutrition, inadequate water, sanitation and hygiene (WASH) conditions and intestinal parasitic infections are intricately linked. Severe malnutrition in school-aged children has been documented in association with inadequate sanitation, poor hygiene and improper child feeding practices [7]. Inadequate WASH conditions are also important risk factors for intestinal parasitic infections that are transmitted through the faecal-oral route [8, 9]. Parasitic infections contribute to stunting by loss of appetite, diarrhoea, mal-absorption and/or an increase in nutrient wastage [10, 11]. Furthermore, infections with intestinal parasites may cause internal bleeding, leading to a loss of iron and anaemia [12], exacerbate the effects of malnutrition, and hence, compromise the development of cognitive abilities [10]. An inadequate dietary intake could lead to weakened immunity, weight loss, impaired growth and increased susceptibility to intestinal parasitic infections [10]. Hence, it is crucial to consider the inter-linkages of malnutrition, intestinal parasitic infections, and WASH for preventive action.

In Nepal, studies related to the inter-linkage of WASH, health and nutrition interventions focusing on increased knowledge and consumption of adequate diet, especially fruits and vegetables, are limited. Efforts to control malnutrition were predominantly targeted to children under the age of 5 years [13]. Deworming campaigns are mainly focussing on school-aged children; however, drug therapy alone might be only a short-term measure for reducing parasitic worm burden among the target population [14]. It has been shown that the prevalence of intestinal parasitic infection returns to the pre-treatment levels within 6 to 18 months after treatment cessation [15–17]. A school garden programme with integrated nutrition education, health and WASH interventions, and increasing knowledge about diet diversity, could address the underlying determinants of nutritional and health problems among school-aged children [18].

A multi-country, multi-sectorial project entitled “Vegetables go to School: improving nutrition through agricultural diversification” (VgtS) was developed and implemented in five countries of Asia and Africa (Bhutan, Burkina Faso, Indonesia, Nepal and the Philippines) to address school-aged children’s nutrition and health problems in an interdisciplinary approach [19]. The objective of the current study was to evaluate whether a school garden and education programme and a school garden with complementary WASH, health and nutrition interventions would improve nutritional and health indices among school-aged children in two districts of Nepal.

## Methods

### Study design

We undertook a randomised controlled trial in 12 schools. Four schools received a school garden and specific education about fruits and vegetables only (SG). Four schools received school garden, coupled with nutrition, health and WASH interventions (SG+). The remaining four schools did not receive any specific interventions (control schools). The two main impact pathways assessed were whether: (a) children’s knowledge about, and intake of, fruits and vegetables will increase by growing fruits and vegetables in both SG and SG+ which, in turn, will improve their nutritional status; and (b) the prevalence of malnutrition, anaemia and intestinal parasite infections among children in SG+ will be reduced, compared to SG and control schools.

### Interventions

#### School gardens with education component (SG)

The first intervention component consisted of a school garden for the cultivation of nutrient-dense vegetables. Teachers were trained in theoretical and practical skills on how to establish and manage school gardens (e.g. levelling and raising land beds, construction of drainage, plantation and caring by children). The trainings were offered twice for 1 week and conducted by project teams, including representatives from the National Agricultural Research Council (NARC), the Ministry of Health and the Ministry of Education. Teachers received different varieties of vegetable seeds and gardening tools and equipment [20]. The school gardens were set up in April 2015. The second intervention consisted of the development and implementation of a curriculum to teach children about gardening (duration: 23 weeks, mainly theory). Teachers received specific training about the use of curriculum by a local project team. The teaching took place once a week during a 90 min class with an emphasis on learning by doing in the school gardens.

Children’s caregivers were invited to visit the school at least twice a year to receive a briefing about the school garden project. Children received small packets of seeds to grow vegetables at home and teachers visited some of the children’s homes for observation of the garden [20]. Two technical staffs were recruited with a background in agriculture. They monitored the school gardens weekly and provided technical assistance as requested by the students and teachers. Single school gardens produced, on average, about 150 kg of vegetables per school year, which were distributed among the children and teachers [21].

#### School garden and complementary interventions (SG+)

In addition to the school garden programme, complementary WASH, health and nutrition interventions were implemented in four schools. The intervention package included the following components:

- Health promotion activities, such as the development of an educational comic booklet that incorporated information about school gardens, nutrition and WASH targeted to school-aged children. Formative research was conducted with children and their caregivers to develop this booklet.
- Provision of a nutrition booklet and hand-outs, incorporating information for children related to fruits and vegetables. The booklet was developed in collaboration with the health personnel.
- Development of a poster to display information related to nutrition, handwashing and waste management for children.
- Demonstration of adequate handwashing with soap. The demonstration was done by health personnel, delivered to children and their caregivers.
- Developing songs related to sanitation and hygiene. Teachers, in collaboration with local authorities, drafted the songs in the schools.
- Audio-visual aids related to nutrition and WASH for children and their caregivers.
- Construction of at least three latrines per school and six to 12 handwashing facilities with the weekly provision of soap (50 bars per week).
- Weekly health education programmes related to nutrition and WASH for caregivers and community stakeholders with the distribution of soap once a week over a 5-month period.
- Organisation of informative sessions for caregivers to explain the school garden programme, highlighting the importance of school gardening and replicating the learnt gardening skills at home to set up home gardens.

These interventions were implemented in combined classes with health education. They were intended to be implemented over a 12-month period. However, due to a major earthquake and a series of aftershocks that hit Nepal in April and May 2015, the duration was abbreviated.

### Study sites, study population and sample size

This study was conducted in the Dolakha and Ramechhap districts. Dolakha is located approximately 180 km and Ramechhap approximately 150 km from Kathmandu, the capital of Nepal. The study population consisted of school-aged children aged 8–17 years at the baseline survey. A Monte Carlo simulation showed that 800 children, with 50 children per school and four schools per intervention arm would provide at least 75% power for finding simultaneous significant effects of the two implemented type of interventions under the following assumptions:

- the prevalence of intestinal protozoan and helminth infections is about 30% [19] and remains constant in the absence of any intervention;
- the probability of new intestinal protozoa and helminth infections at follow-up is 10%;
- the same effect odds ratios (ORs) apply to incidence and persistence of intestinal protozoa and helminth infection; and
- each of the two interventions reduces the odds of infection by 50%, and their effects are additive on the logit-scale.

**Fig. 1:**
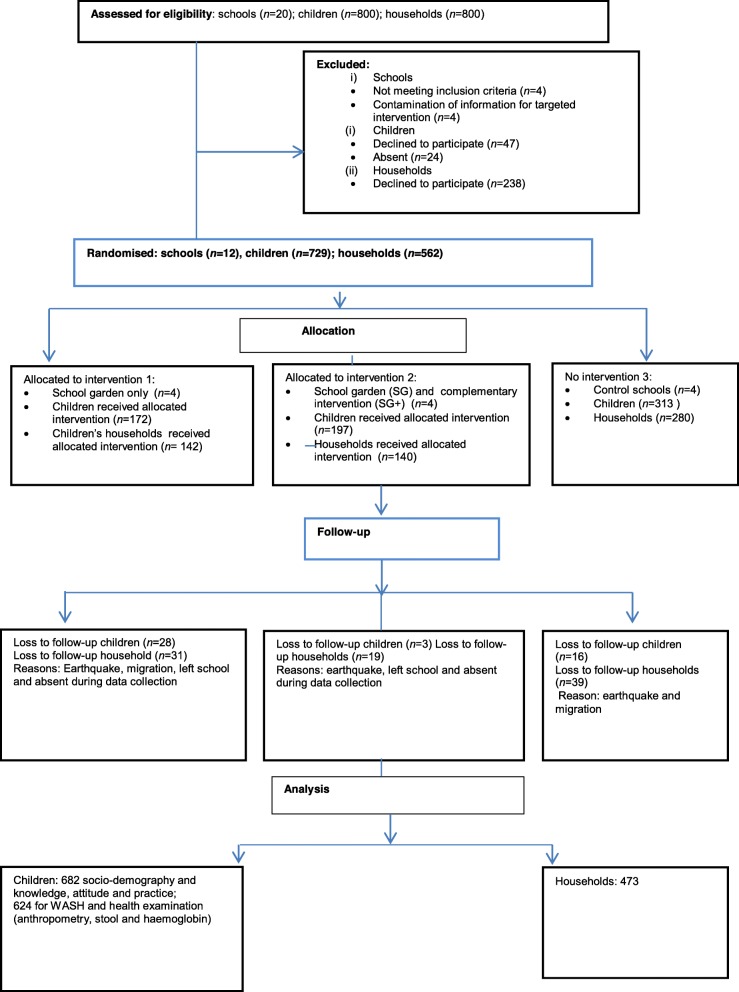
Study 1 compliance of the study population

The study was registered as a cluster randomised controlled trial with study ID ISRCTN17968589 (date assigned: 17 July 2015). The study intended to measure and compare the impact of SG and SG+ interventions on school-aged children’s nutritional and health indices in comparison to control schools. At baseline, a total of 12 schools (10 in Dolakha and two in Ramechhap) were selected randomly among 30 schools that met the following inclusion criteria: (a) schools located within one-hour walking distance from a main tarmac road; and (b) water available at school for vegetable cultivation. Only two schools were included in the Ramechhap district, as the two criteria were difficult to meet. The schools were then randomly allocated to one of the three study arms (Fig. 1)*.* In the first arm, schools received the school garden and education component about gardening only (SG); in the second arm they additionally received WASH, health and nutrition interventions (SG+), while no specific interventions were implemented in the third arm; hence, serving as control. The details of the study protocol have been published elsewhere [19].

### Outcome indicators

The outcome indicators and expected results are presented in Table 1*.* The presented outcomes were based on the project’s impact pathway that assumes stepwise changes in the children’s knowledge of fruits and vegetables and intake via school garden that might lead to a change in children’s nutritional and health status.

**Table 1:** Outcome indicators and expected results among schoolchildren in three intervention arms (SG, SG+ and control) in a randomised controlled trial conducted in two districts of Nepal between March 2015 and June 2016

Outcome	Description of outcome	Expected results
Outcome 1 (Primary outcome)	Change in knowledge about fruits and vegetables, malnutrition, anaemia and intestinal parasitic infection	Schoolchildren know about:
		• the average daily requirement of intake of fruits and vegetables
		• malnutrition and its causes
		• importance of consuming fruits and vegetables for improved health
		• WASH and related diseases including intestinal parasitic infections
Outcome 2	Change in dietary diversity and fruits and vegetables intake	• the dietary diversity score (DDS) and the average fruits and vegetables consumption will increase among school children among SG+
		• the dietary behaviour translates into behaviour change towards increased fruits and vegetables consumption
Outcome 3	Change in nutritional status and haemoglobin level	• the improvement in children’s weight and height among schoolchildren in the SG+ arm • the increase of blood haemoglobin levels among schoolchildren in the SG+ arm
Outcome 4 (Primary outcome)	Change in intestinal parasitic infection	• the incidence of intestinal parasitic infections among schoolchildren from intervention schools will be decreased
Outcome 5	Change in water quality, sanitation and hygiene conditions	• WASH conditions will be improved with well-tailored package of interventions implemented at the unit of schools and households

### Data collection procedures

The same instruments were employed in the baseline and end-line surveys (Additional file 1, Additional file 2, Additional file 3 and Additional file 4). The school directors, district and village authorities, parents and children were informed about the purpose and procedures of the study. Enumerators with a background including higher secondary education and health sciences were recruited for a questionnaire survey. The enumerators were not involved in the implementation of the project and were blinded to the intervention status of the school. Written informed consent was obtained from the children, parents or legal guardians of the children. The voluntary nature of participation in the research activities was emphasised. Children aged 8–17 years were enrolled at baseline. At the follow-up survey in June 2016, the same children were re-assessed. Each child was given a unique identification code for the different assessments at the onset of the study.

The sampled children provided fresh mid-morning, post-exercise stool sample, which were processed and analysed the same day by using the Kato-Katz technique, a formalin-ether concentration and a saline wet mount concentration method. The intensity of infection was calculated as the number of eggs per gram of stool (EPG). The selected school-aged children were subjected to anthropometric measurements according to standard operating procedures, as described by the World Health Organization (WHO), using a digital scale and a height measuring board with a precision of 0.1 kg and 0.1 cm, respectively. The haemoglobin (Hb) level was measured and used as a proxy for anaemia, using a Hemocue portable device (HemoCue Hb 201+ System; HemoCue AB, Angelholm, Sweden). Drinking water samples were collected at the unit of schools, households and community water source [22]. The water samples were analysed in situ at the schools and households for turbidity, pH, chlorine residuals and microbial quality using the DelAgua Kit (Oxfam-DelAgua; Guildford, UK) using readily available standard operating procedures. Details of the data collection procedure are described in a previously published study protocol [19].

### Statistical analysis

Data were described using percentages, frequencies and means. To characterise household socioeconomic status, we conducted a factor analysis to group households into three socioeconomic strata from a list of 18 household assets and construction material of the house wall, roof and floor [23]. Three factors reflecting household socioeconomic status were retained and each of them divided into three strata (high, middle and poor) using the k-means procedure. The data were analysed according to the intention to treat principle. As the children who are symptomatic at baseline often systematically differ from children who were asymptomatic at baseline, we decided to not just study change in prevalence but to distinguish change in children who were asymptomatic (i.e. by studying incidence) and change in children who were symptomatic (i.e. by studying “remission” or “persistence” which equals “1-remission”). Mixed logistic regression models with random intercepts of schools adjusting for age, sex, socioeconomic status and districts were used to estimate intervention effects on incidence and persistence of binary outcomes, such as intestinal parasite infections, anaemia, stunting and thinness, between baseline and end-line. These models also included the factors district, sex and age group of children, and socioeconomic status. To address change in prevalence, repeated measures analyses with additional random intercepts at the level of children were used. Models of change in prevalence involved group-specific indicator variables for end-line observations along with indicator variables for the two follow-up groups, to obtain group-specific ORs of change in prevalence. The statistical significance of the differences between these ORs in the intervention groups and the respective ORs in the control group was determined by replacing the end-line indicator variable of the control group by the overall end-line indicator variable. To address potential period effects and interactions of this variable with the intervention indicator variables to estimate and compare changes in prevalence across the different study arms. The change in prevalence is determined by the persistence (e.g. children who were stunted at baseline and were still stunted at end-line and whether there was a difference between groups) and incidence along with the baseline prevalence according to the formula:

$$ \mathrm{Prevalence}\ \mathrm{at}\ \mathrm{follow}-\mathrm{up}=\left(\mathrm{prevalence}\ \mathrm{at}\ \mathrm{baseline}\right)\ast \mathrm{persistence}+\left(1-\mathrm{prevalence}\ \mathrm{at}\ \mathrm{baseline}\right)\ast \mathrm{incidence} $$

All effect estimates regarding dichotomous outcomes are reported as ORs with 95% confidence intervals (CIs).

Mixed linear regression models with random intercepts for schools adjusting for age, sex of the children, socio-economic status of the caregivers and districts were applied to assess intervention effects on longitudinal changes of continuous variables such as dietary diversity scores (DDS), height and weight, and Hb level. These models included the baseline value of the respective outcome as one of the predictor variables along with age, sex, district and socioeconomic status. Differences were considered statistically significant if *p*-values were < 0.05. All analyses were carried out using STATA, version 14 (STATA Corporation; College Station, TX, USA).

## Results

### Study compliance and characteristics of study population

Of the 708 children who were enrolled at the March 2015 baseline survey, 682 children completed the questionnaire survey and 624 children completed all aspects of health and nutritional examination (anthropometry, stool examination and, Hb measurements) at the June 2016 end-line survey. Of four schools allocated to receive the SG intervention, a total of 172 children completed the follow-up. For the four schools allocated to receive the SG+, a total of 197 children completed the end-line and for the four schools allocated to the control group without any intervention, 313 children completed the end-line survey in both districts. Due to the proximity of the earthquake epicentre to the study area, which destroyed around 75% of schools and households in May 2015, 26 children were lost from baseline and 89 of 562 households were no longer accessible at the end-line survey in both districts. Hence, complete data were available from 433 households. Therefore, the final analysis included 433 households, 682 schoolchildren for socio-demography and knowledge, attitude and practice (KAP) and 624 for clinical examination (anthropometry, stool and Hb) (Fig. 1). We compared the baseline socioeconomic status of the households having participated in the follow-up with those households which were lost to follow-up. From the 31.2% households classified with a high socioeconomic status at baseline, only 8.7% remained in this class at end-line. The percentage of households with an average socioeconomic status increased from 30.9 to 38.3%, while households with poor socioeconomic status increased from 37.9 to 53.0% over the 15-month study period.

The characteristics (e.g. sex and age) of children and caregivers who completed the follow-up study are described in Table 2. More than half of the surveyed children were boys (52.7%). There was substantial heterogeneity in the educational status of caregivers across study arms, with 51.4% of caregivers being without formal education in SG+ compared to 26.6% in the control arm, which has also been taken into account in the statistical analysis. The primary occupation of caregivers was farming across all study arms (90.7% in SG+, 79.3% in SG and 78.1% in control; *p* < 0.01). More than three quarter of the school-aged children from all groups had domestic animals in their households (85.0% SG+, 86.8% SG and 94.0% control; *p* < 0.01). Most of school-aged children’s households had agricultural land (82.9% SG+, 92.7% SG and 94.0% control; *p* < 0.01), and the self-food production was slightly lower in the SG+ arm (82.1%; compared to 90.1% in SG and 91.0% in control; *p <* 0.01).

**Table 2:** Characteristics of schoolchildren and caregivers in Dolakha and Ramechhap districts, Nepal, at baseline, March-May 2015

Characteristics	Control	SG-intervention^a^	Combined intervention (SG+)^b^	Total	*p-value*
	[n, (%)]	[n, (%)]	[n, (%)]		
Children’s demographic characteristics					
Sex					
Female	156 (47.3)	77 (43.0)	106 (53.3)	339 (47.9)	0.13
Male	174 (52.7)	102 (57.0)	93 (46.7)	369 (52.1)	
Age groups					
Age group 1 (8–12 years)	47 (14.2)	29 (16.2)	32 (16.1)	108 (15.3)	0.78
Age group 2 (13–17 years)	283 (85.8)	150 (83.8)	167 (83.9)	600 (84.7)	
Caregivers demographic characteristics					
Caregivers education					
No formal schooling	80 (26.6)	58 (48.0)	72 (51.4)	210 (37.4)	**< 0.01**
Primary education	72 (24.0)	36 (29.8)	36 (25.7)	144 (25.6)	
Secondary education	94 (31.2)	22 (18.2)	27 (19.3)	143 (25.4)	
Higher education	55 (18.3)	5 (4.1)	5 (3.4)	65 (11.6)	
Caregivers ethnicity					
Brahmin	28 (9.3)	52 (37.1)	21 (17.4)	101 (18.0)	**< 0.01**
Chhetri	102 (33.9)	56 (46.3)	52 (37.1)	210 (37.4)	
Newar	15 (5.0)	14 (11.6)	4 (2.9)	33 (5.9)	
Tamang	152 (50.5)	30 (24.8)	31 (22.1)	213 (37.9)	
Janajati	4 (1.3)	0 (0.0)	1 (0.7)	5 (0.9)	
Caregivers occupation					
No occupation	21 (7.0)	0 (0.0)	4 (2.9)	25 (4.5)	**< 0.01**
Farmer	235 (78.1)	96 (79.3)	127 (90.7)	458 (81.5)	
Public service	17 (5.7)	17 (14.1)	5 (3.6)	39 (6.9)	
Business owner	28 (9.3)	8 (6.6)	4 (2.9)	40 (7.1)	
Socioeconomic characteristics					
Roof materials					
Corrugated iron roof	272 (90.4)	59 (48.8)	84 (60.0)	415 (73.8)	**< 0.01**
Wood and tiles	29 (9.6)	62 (51.2)	56 (40.0)	147 (26.2)	
Wall materials					
Wood	41 (13.6)	15 (12.4)	10 (7.1)	66 (11.7)	**0.05**
Corrugated iron	47 (15.6)	12 (9.9)	30 (21.4)	89 (15.8)	
Bricks	213 (70.8)	94 (77.7)	100 (71.4)	407 (72.4)	
Floor materials					
Mud	270 (89.7)	115 (95.0)	139 (99.3)	524 (93.2)	**< 0.01**
Cement	31 (10.3)	6 (5.0)	1 (0.7)	38 (6.8)	
Energy for cooking					
Charcoal/wood	254 (84.4)	96 (79.3)	123 (87.9)	473 (84.2)	0.17
Electricity	47 (15.6)	25 (20.7)	17 (12.1)	89 (15.8)	
Socioeconomic status					
High	28 (9.3)	15 (12.4)	6 (4.3)	49 (8.7)	**< 0.01**
Middle	96 (31.9)	62 (51.2)	57 (40.7)	215 (38.3)	
Poor	177 (58.8)	44 (36.4)	77 (55.0)	298 (53.0)	
Own agricultural land	283 (94.0)	112 (92.7)	116 (82.9)	511 (90.9)	**< 0.01**
Total production					
≤ 10%	13 (4.3)	9 (7.4)	22 (15.7)	44 (7.8)	**< 0.01**
10–30%	14 (4.7)	3 (2.5)	3 (2.1)	20 (3.6)	
≥ 30%	274 (91.0)	109 (90.1)	115 (82.1)	498 (88.6)	
Possession of domestic animals	283 (94.0)	105 (86.8)	119 (85.0)	507 (90.2)	**< 0.01**

Socioeconomic status was derived from a factor analysis of variables indicating the possession of household assets such as a radio, a television, a mobile phone, a table, a stove, a petrol lamp, a motorbike, a car or truck, a watch, an iron, a bike, a cupboard etc. The score of the first factor was then divided into three categories using the k-means procedure

^a^SG School garden intervention

^b^SG+ School garden, nutrition and water, sanitation and hygiene (WASH) interventions

*p*-values were obtained by *χ*^2^ test

### Outcomes 1 and 2: change in knowledge about fruits and vegetables, dietary diversity, malnutrition, anaemia and intestinal parasitic infection

The changes in key indicators from the questionnaire related to knowledge about fruits and vegetables, malnutrition, anaemia and intestinal parasitic infections in the surveyed school-aged children’s households are presented in Table 3.

**Table 3:** Change in knowledge about fruits and vegetables, malnutrition, anaemia, intestinal parasitic infections and dietary diversity at baseline and during follow-up across the different study arms in Dolakha and Ramechhap districts, Nepal (March–May 2015 and June 2016)

Nutrition variables	Categories	Control		SG-intervention (SG)		Combined intervention (SG+)		Effect of SG-intervention (95% CI)	*p*-value	Effect of combined intervention (95% CI)	*p*-value
		Baseline (*n* = 313)	End-line (*n* = 313)	Baseline (*n* = 172)	End-line (*n* = 172)	Baseline (197)	End-line (*n* = 197)				
Self-reported daily requirement of frequency of fruit and vegetable consumption^a^	0	34 (10.9)	0 (0.0)	29 (16.9)	0 (0.0)	17 (8.6)	0 (0.0)	0.15 (−0.33–0.63)^c^	0.55	0.15 (−0.32–0.63)^c^	0.53
	1	7 (2.2)	64 (20.5)	7 (4.1)	25 (14.5)	11 (5.6)	28 (14.2)				
	2	20 (6.4)	166 (53.0)	18 (10.5)	98 (57.0)	19 (9.6)	120 (60.9)				
	3	117 (37.4)	0 (0.0)	68 (39.5)	0 (0.0)	98 (49.8)	0 (0.0)				
	4	101 (32.3)	0 (0.0)	29 (16.9)	0 (0.0)	38 (19.3)	0 (0.0)				
	≥5	34 (10.9)	83 (26.5)	21 (12.2)	49 (28.5)	14 (7.1)	49 (24.9)				
Opinion about fruits and vegetables consumptions^a,b^	0	24 (7.7)	27 (8.6)	15 (8.7)	11 (6.4)	20 (10.1)	3 (1.5)	0.07 (−0.12–0.25)^c^	0.48	0.21 (0.02–0.39)^d^	**0.03**
	1	58 (18.5)	12 (3.8)	61 (35.5)	0 (0.0)	38 (19.3)	0 (0.0)				
	2	231 (73.8)	274 (87.5)	96 (55.8)	161 (93.6)	139 (70.6)	194 (98.5)				
Consumption of green vegetables prior to day of survey		123 (39.3)	177 (56.5)	50 (29.1)	98 (57.0)	87 (44.2)	102 (51.8)	0.70 (0.10–4.86)^d^	0.72	0.76 (0.10–5.89)-^d^	0.80
Heard about malnutrition		83 (26.5)	213 (68.0)	44 (25.6)	122 (70.9)	87 (44.2)	174 (88.3)	1.48 (0.85–2.57)^d^	0.17	6.08 (3.01–12.3)^d^	**< 0.001**
Perception of malnutrition as a problem		67 (21.4)	189 (88.7)	34 (19.8)	115 (94.3)	73 (37.1)	165 (94.3)	2.19 (0.26–18.6)^d^	0.47	2.51 (0.34–18.5)^d^	0.37
Responses related to the causes of malnutrition	Disease	0 (0.0)	44 (14.1)	0 (0.0)	3 (1.7)	2 (1.0)	5 (2.5)	0.09 (0.005–1.58)^d^	0.10	0.12 (0.01–1.43)^d^	0.09
	Lack of food	19 (6.1)	95 (30.3)	11 (6.4)	36 (20.9)	11 (5.6)	63 (32.0)	2.53 (0.47–13.5)^d^	0.28	1.10 (0.20–5.95)^d^	0.91
	Irregular meal	19 (6.1)	108 (34.5)	14 (8.1)	46 (26.7)	30 (15.2)	83 (42.1)	2.06 (0.49–8.62)^d^	0.32	0.36 (0.08–1.77)^d^	0.21
	Poorly prepared food	2 (0.6)	47 (15.0)	1 (0.6)	14 (8.1)	3 (1.5)	19 (9.6)	0.28 (0.08–1.02)^d^	**0.05**	0.80 (0.26–2.45)^d^	0.70
	Lack of means to afford good food	3 (1.0)	36 (11.5)	0 (0.0)	10 (5.8)	5 (2.5)	24 (12.2)	0.58 (0.12–2.75)^d^	0.50	1.90 (0.45–8.10)^d^	0.39
Heard about anaemia		128 (63.4)	122 (60.1)	49 (24.3)	36 (17.7)	25 (12.4)	45 (22.2)	0.52 (0.27–1.00)^d^	**0.05**	0.46 (0.24–0.87)^d^	**0.02**
Heard about night blindness		126 (55.7)	156 (54.2)	62 (27.4	71 (24.6)	38 (16.8)	61 (21.2)	0.98 (0.23–4.07)^d^	0.97	0.52 (0.13–2.13)^d^	0.36
Heard about intestinal parasitic infections		50 (37.6)	199 (57.3)	42 (31.6)	66 (19.0)	41 (30.8)	82 (23.6)	0.26 (0.07–0.92)^d^	**0.04**	0.68 (0.18–2.63)^d^	0.58
Dietary diversity score^a^	1	2 (0.6)	28 (9.0)	2 (1.2)	24 (14.0)	0 (0.0)	14 (7.1)	−0.67 (−1.58–0.24)^c^	0.15	−0.30 (−1.22–0.63)^c^	0.53
	2	30 (9.6)	33 (10.5)	26 (15.1)	27 (15.7)	11 (5.6)	25 (12.7)				
	3	106 (33.9)	32 (10.2)	53 (30.8)	21 (12.2)	50 (25.4)	21 (10.7)				
	4	109 (34.8)	29 (9.3)	53 (30.8)	21 (12.2)	72 (36.6)	22 (11.2)				
	5	50 (16.0)	30 (9.6)	32 (18.6)	22 (12.8)	49 (24.9)	29 (14.7)				
	6	15 (4.8)	27 (8.6)	5 (2.9)	20 (11.6)	14 (7.1)	29 (14.7)				
	7	1 (0.3)	29 (9.3)	0 (0.0)	13 (7.6)	1 (0.5)	34 (17.3)				
	8	0 (0.0)	44 (14.1)	1 (0.6)	13 (7.6)	0 (0.0)	19 (9.6)				
	9	0 (0.0)	61 (19.5)	0 (0.0)	11 (6.4)	0 (0.0)	4 (2.0)				

*SG* School garden

SG+ School garden, nutrition, health and water, sanitation and hygiene (WASH)

^a^These variables were treated as numerical in the analysis

^b^Here, “0” = It is not good, “1” = I am not sure and “2” = It is good

^c^Intervention effects were estimated by mixed linear models for the respective end-line outcome, including the factor group and random intercepts for the schools, while also adjusting for the outcome observed at baseline, the district, sex and age of the child, and education level and socioeconomic status of the caregivers. The effect estimates can be interpreted as adjusted differences in the mean changes of the respective variables between the given intervention group and the control group

^d^Odds ratio of desired follow-up outcome between the respective intervention group and the control group from a mixed logistic regression model, including the factor group and random intercepts for the schools, while also adjusting for the outcome observed at baseline, the district, sex and age of the child, and education level and socioeconomic status of caregivers

An increase of knowledge regarding the importance of consuming ≥5 portion of vegetables and fruits per day was found mostly among SG+ school-aged children (7.1 to 24.9% in SG+, 12.2 to 28.5% in SG and 10.9 to 26.5% in control). The improvement in knowledge about requirement of vegetables in diet also translated into behavioural change by increasing in the intake of vegetables, i.e. SG+ (33.5 to 74.6%), SG (37.2 to 74.4%) and control arm (33.9 to 77.0%). The proportion of households preparing vegetables increased in all three arms (from 70.2 to 95.0%, in SG+, from 81.1 to 86.5%, in SG and from 91.3 to 93.7% in the control arm). The same was true for the proportion of households giving fruits to children (from 49.0 to 51.0% in SG+, from 50.4 to 14.2% in SG and from 54.6 to 76.6% in the control arm).

Similarly, the percentage of school-aged children who heard about malnutrition increased in all schools, but most strongly in SG+ (44.2 to 88.3%), followed by SG (25.6 to 70.9%) and the control arm (26.5 to 68.0%). The same was true for the proportion of children who heard about anaemia, which increased most strongly in SG+ (12.4 to 22.4%) in comparison to SG (24.3 to 17.7%), while there was a slight decrease in control schools (63.4 to 60.1%). In contrary, children who heard about intestinal parasitic infection increased in control (37.6 to 57.3%) in comparison to SG (31.6 to 19.0%) and SG+ schools (30.8 to 23.6%).

### Outcome 3: changes in anthropometric indicators and anaemia among school-aged children

The changes in anthropometric indicators and anaemia among school-aged children are shown in Table 4. Stunting was slightly lowered in SG+ (19.9 to 18.3%) and in the control arm (19.7 to 18.9%) and slightly increased in SG (17.7 to 19.5%), however, without a statistically significant difference. Thinness increased both in SG+ (5.7 to 9.9% compared to control) and SG (9.7 to 10.4% compared to control) and decreased in the control arm (12.3 to 7.1%). There was a slight reduction in anaemia in SG+ (33.0 to 32.0%) but a major increase was observed in SG (20.7 to 43.9%) and the control arm (22.7 to 41.3%).

**Table 4:** Odds ratios of change in prevalence from baseline to end-line for parasitic infections, anaemia, stunting and thinness, in a cohort of schoolchildren in two districts of Nepal, March-May 2015 and June 2016

Outcomes	Group	Baseline prevalence (%)	End-line prevalence (%)	Odds ratio^a^ (OR)	95% CI	p-value^**^
Stunting^b^	Control	19.7	18.9	0.91	0.56–1.49	
	SG-intervention	17.7	19.5	1.17	0.62–2.20	0.54
	Combined intervention (SG+)	19.9	18.3	0.88	0.49–1.56	0.92
Thinness^b^	Control	12.3	7.1	0.47	0.24–0.94	
	SG-intervention	9.7	10.4	1.09	0.48–2.48	0.12
	Combined intervention (SG+)	5.7	9.9	2.10	0.88–5.02	**< 0.01**
Anaemia^c^	Control	22.7	41.3	3.06	1.97–4.77	
	SG-intervention	20.7	43.9	3.77	2.17–6.56	0.56
	Combined intervention (SG+)	33.0	32.0	0.94	0.59–1.51	**< 0.01**
Intestinal parasitic infections	Control	43.9	42.4	0.95	0.67–1.36	
	SG-intervention	33.5	27.4	0.75	0.46–1.20	0.42
	Combined intervention (SG+)	37.1	9.4	0.16	0.09–0.29	**< 0.01**

*SG* School garden

SG+ School garden, nutrition, and water, sanitation and hygiene (WASH)

**The *p*-value refers to the difference between the respective odds ratio and the corresponding odds ratio in the control group

^a^Odds ratios of change in prevalence were estimated by mixed logistic regression models for symptom status at baseline and end-line, including the factor group and group-specific indicator variables for endline measurements, as well as random intercepts for schools and for children. Further adjustment was made for the district, age and sex of the child, and for education level of caregivers

^b^Stunting: height for age < −2 SD of the WHO Child Growth Standards Median

^b^Thinness: weight for height < −2 SD of the Child Growth Standards Median

^c^Cut-off point for anaemia: haemoglobin lower than 80 g/l

The persistence and incidence of anthropometric indicators and anaemia at end-line are shown in Table 5. The persistence of stunting was slightly lower in SG+ (36.8%) than in the control arm (37.7%). The incidence of stunting was slightly higher in SG (16.3%) than SG+ (13.7%) and control arm (14.3%). The mean increase in height and weight were highest in SG+ (6.8 cm and 5.8 kg, respectively) and the control schools (5.2 cm and 6.2 kg, respectively) and considerably lower in SG (3.2 cm and 3.5 kg, respectively). The height and weight gains in the SG arm were significantly lower than the ones in the control arm. Persistence of anaemia was higher in SG (67.6%) than in SG+ (47.6%) and the control arm (52.5%). The mean change in Hb level was significantly higher in SG+ than in the control arm (∆ = 0.58, 95% CI: − 0.26-1.43; *p* = 0.18).

**Table 5:** Changes of nutritional indicators in the study cohort by group (control, intervention (SG) and additive Intervention (SG+)) in two districts of Nepal, June 2016

Outcomes	End-line (June 2016)			Effect of SG-intervention (95% CI)^a^	*p-value*	Effect of combined intervention (SG+) (95%CI)^a^	*p-value*
	Control (%)	SG-intervention (%)	Combined intervention (SG+) (%)				
Logistic models (binary outcomes)							
Persistence^b^ of stunting	20 (37.7)	10 (34.5)	14 (36.8)	0.57 (0.13–2.60)	0.47	0.78 (0.18–3.25)	0.73
Persistence of thinness	8 (24.2)	4 (25.0)	4 (36.4)	1.36 (0.22–8.29)	0.74	3.50 (0.43–28.74)	0.24
Persistence of overweight	4 (66.7)	0 (0.0)	1 (14.3)	n/a		n/a	
Persistence of anaemia	32 (52.5)	23 (67.6)	30 (47.6)	2.21 (0.74–6.62)	0.15	0.70 (0.27–1.81)	0.46
Incidence of stunting	31 (14.3)	22 (16.3)	21 (13.7)	0.60 (0.28–1.29)	0.19	0.60 (0.24–1.49)	0.27
Incidence of thinness	11 (4.70)	13 (8.8)	15 (8.3)	2.06 (0.81–5.21)	0.13	3.07 (1.10–8.61)	**0.03**
Incidence of overweight	14 (5.3)	6 (3.7)	6 (3.3)	0.38 (0.86–1.65)	0.20	0.58 (0.13–2.61)	0.48
Incidence^c^ of anaemia	79 (38.0)	49 (37.7)	31 (24.2)	1.20 (0.43–3.33)	0.73	0.56 (0.16–1.90)	0.35
Linear model (continuous outcomes)^d^							
Change in height-for-age (for stunting)	−0.02 (−0.24, 0.19)	−0.16 (0.44, 0.13)	0.19 (−0.09, 0.46)	0.05 (−0.46–0.56)	0.85	0.41 (−0.17–0.98)	0.41
Change in BMI-for age (for thinness)	1.58 (1.14, 2.02)	0.94 (0.35, 1.54)	1.02 (0.44, 1.59)	−0.98 (−1.74- (− 0.22))	0.01	− 0.64 (−1.49–0.22)	0.14
Height gain (cm)	5.20 (3.98, 6.43)	3.20 (1.59, 4.81)	6.84 (5.33, 8.35)	−0.52 (−2.67–1.63)	0.64	2.88 (− 0.54–5.23)	**0.02**
Weight gain (kg)	6.16 (5.11, 7.21)	3.50 (2.09, 4.91)	5.75 (4.42, 7.09)	−2.15 (−4.55- (−0.26))	**0.08**	0.22 (− 2.47–2.92)	0.87
Change in haemoglobin level (g/dl)	−0.64 (− 0.98, − 0.30)	−0.60 (− 1.06, − 0.13)	−0.03 (− 0.48, 0.42)	−0.03 (− 0.78–0.72)	0.94	0.58 (− 0.26–1.43)	0.18

*SG* School garden

SG+ School garden, nutrition, and water, sanitation and hygiene (WASH)

^a^Logistic regression models: odds ratio from a mixed logistic regression model of outcome at follow-up as a function of outcome at baseline with a random effect for school adjusting for socioeconomic status

^b^Persistence: Children who were still symptomatic at follow-up (i.e. did not have a remission)

^c^Incidence: Occurrence of new cases

^d^First three columns contain estimates and 95% confidence intervals of average changes from baseline to follow-up in the respective study arms, obtained using mixed linear regression models with random intercepts at the level of schools. Column 4 to 7 contain intervention effects (SG vs control and SG+ vs control) estimated by mixed linear models for the changes in the respective outcome variables, with the two intervention indicator variables as main predictors and random intercepts for the schools, further adjusting for the district, age and sex of the child, and socioeconomic status of the caregivers. Estimates can be interpreted as adjusted differences in the mean changes of the respective variables between the given intervention group and the control group

### Outcome 4: change in intestinal parasitic infections in school-aged children

At baseline, the prevalence of intestinal parasitic infections, among school-aged children in the three arms, were all high (37.1% in SG+, 33.5% in SG, and 43.9% in the control arm). At the end-line, there was a strong decline to 9.4% in SG+, while the prevalence showed only minor changes in SG and the control arms (Table 4).

The persistence and incidence of intestinal parasitic infections at the end-line are presented for all study arms in Table 6. The persistence of overall intestinal parasitic infections was significantly lower in SG+ than in the control arm (8.4% vs. 45.8%, *p* < 0.01). The incidence of overall intestinal parasitic infections was highest in the control arm (39.7%), intermediate in SG (25.7%, *p* = 0.07 compared to the control arm) and lowest in SG+ (10.0%, *p* < 0.01 compared to the control arm). The persistence of overall intestinal protozoa infection was lowest in SG+ (0.0%), comparable in SG (9.1%) and the control arm (10.3%). Similarly, the incidence of overall intestinal protozoa infection was lowest in SG+ (1.5%, *p* = 0.03 compared to control), intermediate in SG (5.8%, *p =* 0.24 compared to control) and highest in the control arm (10.4%). Similar patterns were observed for the persistence (a) and incidence (b) of overall soil-transmitted helminth infections, with values for (a) of 10.3% (SG+), 28.3% (SG) and 47.5% (control arm), and for (b) of 7.3% (SG+), 18.0% (SG) and 28.5% (control arm).

**Table 6:** Intestinal parasitic infections change during follow-up across the different study arms in Dolakha and Ramechhap districts, Nepal (March-May 2015 versus June 2016)

Outcomes	End-line (June 2016)			Effect of SG-intervention (95% CI)^b^	*p-value*	Effect of combined intervention (SG+) (95% CI)^b^	*p-value*
	Control (*n* = 151/118)^a^ (%)	SG-intervention (*n* = 109/55)^a^ (%)	Combined intervention (SG+) (*n* = 120/71)^a^ (%)				
Persistence of overall intestinal parasitic infections	54 (45.8)	17 (30.9)	6 (8.4)	0.71 (0.30–1.69)	0.44	0.14 (0.01–0.68)	**< 0.01**
Persistence of overall intestinal protozoa infection	9 (10.3)	4 (9.1)	0 (0.0)	0.69 (0.15–3.25)	0.64	n/a	n/a
Persistence of overall soil-transmitted helminth infections	56 (47.5)	15 (28.3)	7 (10.3)	0.54 (0.21–1.41)	0.21	0.20 (0.05–0.82)	**0.03**
Persistence of overall nematode infections	53 (46.1)	11 (22.0)	7 (11.1)	0.34 (0.12–0.94)	**0.04**	0.23 (0.06–0.91)	**0.04**
Incidence of overall intestinal parasitic infections	60 (39.7)	28 (25.7)	12 (10.0)	0.48 (0.22–1.05)	0.07	0.09 (0.03–0.28)	**0.01**
Incidence of overall intestinal protozoa infections	19 (10.4)	7 (5.8)	2 (1.5)	0.55 (0.20–1.50)	0.24	0.11 (0.01–0.84)	**0.03**
Incidence of overall soil-transmitted helminth infection	43 (28.5)	20 (18.0)	9 (7.3)	0.49 (0.18–1.31)	0.15	0.05 (0.01–0.30)	**< 0.01**
Incidence of overall nematode infections	39 (25.3)	15 (13.2)	9 (7.0)	0.31 (0.08–1.13)	0.08	0.06 (0.01–0.43)	**< 0.01**

Persistence was analysed in the sample of children who had the outcome at baseline and incidence among children who were free of the outcome at baseline

*SG* School garden

SG+ School garden, nutrition, and water, sanitation and hygiene (WASH)

^a^The first number (n) is for the children having been without the parasite at baseline and the second one (n) for children having been infected by the respective parasite at baseline

^b^Odds ratio from a mixed logistic regression model of the outcome at follow-up as a function of the outcome at baseline and type of intervention, with random intercepts for the schools and further adjustment for the district, age and sex of the child, and socioeconomic status of the caregivers

### Outcome 5: changes in drinking water quality in households and KAP on WASH among school-aged children

The thermo-tolerant coliforms (TTC) in the drinking water showed considerably higher percentages in all study groups at the end-line compared to baseline (increase from 0.0 to 13.7% in SG+, increase from 2.4 to 9.5% in SG and increase from 3.9 to 14.8% in the control arm) (Table 7).

**Table 7:** Water quality parameters at baseline and its change during follow-up across the different study arms in Dolakha and Ramechhap districts, Nepal (March-May 2015 and June 2016)

Category	Parameters	Unit	Range	Baseline (March–May, 2015)			End-line (June 2016)			Effect of SG- intervention (95% CI)	*p-value*	Effect of combined intervention (SG+) (95% CI)	*p-value*
				Control n (%)	SG- intervention n (%)	Combined intervention (SG + N + WASH) n (%)	Control n (%)	SG-intervention n (%)	Combined intervention (SG+) n (%)				
Linear model (continuous outcome)													
Physical characteristics	Turbidity	NTU	> 5	7 (3)	3 (4)	0 (0)	46 (20)	26 (31)	23 (32)	0.53		0.58	
			2–5	223 (97)	81 (96)	73 (100)	184 (80)	58 (69)	50 (68)	(−0.70–1.77)	0.4	(−0.82–1.99)	0.42
	pH		6.5–8.5	230 (100)	84 (100)	73 (100)	230 (100)	84 (100)	73 (100)				
Chemical characteristics	Free residual chlorine	mg/l	0.3–0.5	0 (0)	0 (0)	0 (0)	42 (18)	26 (31)	18 (25)	0.02		0.01	
			0.1–0.2	230 (100)	84 (100)	73 (100)	188 (82)	58 (69)	55 (75)	(−0.01–0.06)	0.26	(−0.03–0.05)	0.78
	Total residual chlorine	mg/l	≥0.5	0 (0)	0 (0)	0 (0)	225 (98)	80 (95)	73 (100)	−0.01		0.02	
			0.2–0.49	230 (100)	84 (100)	73 (100)	2 (1)	0 (0)	0 (0)	(−0.03–0.04)	0.7	(−0.02–0.05)	0.35
			0–0.19	0 (0)	0 (0)	0 (0)	3 (1)	4 (5)	0 (0)				
Microbiological characteristics	Thermotole-rant coliforms	CFU/100 ml	0	170 (74)	68 (81)	68 (93)	171 (74)	59 (70)	52 (71)	−5.33		−0.99	
			1–10	37 (16.)	10 (12)	5 (7)	9 (4)	13 (15)	4 (5)	(−34.6–23.9)	0.72	(−33.2–31.3)	0.95
			11–100	14 (6)	4 (5)	0 (0)	16 (7)	4 (5)	7 (10)				
			> 100	9 (4)	2 (2)	0 (0)	34 (15)	8 (10)	10 (14)				

*SG* School garden

SG+: School garden, nutrition and water, sanitation and hygiene (WASH)

*Estimated by a mixed linear regression model for the change in the respective outcome variable, with the two intervention indicator variables as main predictors and random intercepts for the schools, further adjusting for the district, age and sex of the child, and socioeconomic status of caregivers

The change in KAP on WASH among school-aged children is shown in Table 8 and Additional file 5: Table S1. Handwashing with soap (a) before eating and (b) after defection showed stronger increased from baseline to end-line in SG+ compared to the control arm, with (a) 74.1 to 96.9% vs. 78.3 to 84.0% (*p* = 0.01), and (b) 77.2 to 99.0% vs. 78.0 to 91.0% (*p* = 0.15). The proportion of children bringing drinking water from home decreased in the SG+ (21.8 to 11.7%), while it increased in SG (11.0 to 27.3%) and control (11.2 to 43.1%). The intervention had no effect on knowledge related to the diseases such as diarrhoea and cholera.

**Table 8:** Change in KAP on water, sanitation and hygiene (WASH) indicators among a study cohort of school-aged children in two districts of Nepal, March–May 2015 and June 2016

Outcomes	Categories	Baseline (March–May 2015)			End-line (June 2016)			Effect of SG- intervention^a^ (95% CI)	*p-value*	Effect of combined intervention (SG + N + WASH)^a^ (95% CI)	*p-value*
		Control (*n* = 313)	SG- intervention (*n* = 172)	Combined intervention (SG+) (*n* = 197)	Control (*n* = 313)	SG- intervention (*n* = 172)	Combined intervention (SG+) (*n* = 172)				
Handwashing:											
Before eating		244 (78.3)	115 (66.9)	146 (74.1)	263 (84.0)	149 (86.6)	191 (96.9)	1.50 (0.66–3.41)	0.34	9.36 (1.89–46.2)	**0.01**
After playing		186 (59.4)	90 (52.3)	127 (64.5)	203 (64.9)	125 (72.7)	141 (71.6)	0.53 (0.21–1.32)	0.18	1.20 (0.41–3.48)	0.74
After defecation		244 (78.0)	117 (68.0)	152 (77.2)	285 (91.0)	168 (97.7)	195 (99.0)	3.57 (0.30–42.1)	0.31	3.03 (0.66–13.9)	0.15
Children bringing drinking water from home		35 (11.2)	19 (11.0)	43 (21.8)	135 (43.1)	47 (27.3)	23 (11.7)	0.63 (0.13–3.02)	0.56	0.09 (0.01–0.70)	**0.02**
Dirty water causing:	Diarrhoea	196 (62.6)	104 (60.5)	140 (71.1)	281 (89.8)	163 (94.8)	196 (99.5)	1.79 (0.64–5.01)	0.27	0.75 (0.31–1.83)	0.53
	Cholera	36 (11.5)	45 (26.2)	54 (27.4)	313 (100.0)	172 (100.0)	197 (100.0)	1.97 (0.82–4.74)	0.13	1.82 (0.70–4.72)	0.22
	Skin irritation	26 (8.3)	7 (4.1)	14 (7.1)	56 (17.2)	15 (8.7)	6 (3.0)	0.22 (0.05–0.99)	0.05	0.73 (0.11–4.94)	0.75
	Typhus	22 (7.0)	10 (5.8)	9 (4.6)	42 (13.4)	8 (4.6)	12 (6.1)	0.22 (0.02–2.42)	0.22	3.03 (0.18–51.0)	0.44
	Eye irritation	2 (0.6)	0 (0.0)	7 (3.5)	22 (7.0)	1 (0.6)	1 (0.51)	0.12 (0.01–6.21)	0.29	0.74 (0.01–1.09)	0.90
	Worms/parasites	28 (8.9)	16 (9.3)	26 (13.2)	52 (16.6)	29 (16.9)	21 (10.7)	0.84 (0.44–1.59)	0.59	0.46 (0.20–1.07)	0.07

*SG* School garden

SG+ School garden, nutrition and water, sanitation and hygiene (WASH)

^a^Odds ratio from a mixed logistic regression model of the outcome at follow-up, as a function of the outcome at baseline and the two intervention indicators, with random intercepts for the schools and further adjustment for the district, age and sex of the child, and socioeconomic status of caregivers

The changes in key indicators from the questionnaire related to WASH in the surveyed school-aged children’s households are presented in Additional file 5: Table S1. The proportion of water sufficiency increased significantly in SG compared to control (83.8 to 98.2%; *p* = 0.003).

## Discussion

Our study assessed the effects of school gardens and complementary nutrition and WASH interventions on children’s KAP about fruits and vegetables, their dietary diversity, intestinal parasitic infections and nutritional status in the districts of Dolakha and Ramechhap, Nepal within the frame of the VgtS project. Only few studies have investigated an effect of SG and SG+ interventions on children’s nutritional practices, anthropometric indices and intestinal parasitic infections. The novelty of our approach was to assess a number of behavioural, health and nutritional outcome indicators in the frame of an integratrated school garden programme.

### Effects on intestinal parasitic infections, anaemia, anthropometry and KAP on WASH

Our results indicate that the SG+ interventions significantly reduced intestinal parasitic infections in comparison to control schools, which might be partly due to the impact of applied interventions such as increase in knowledge in handwashing before eating and deworming with 6 months intervals. Consistently, the strongest increase in the school-aged children’s handwashing before eating was observed in the SG+ arm. Furthermore, significant improvements in caregivers’ knowledge on nutrition indicators, such as preparation of vegetables and giving fruits to children, increased in the SG+ arm. Stunting was slightly decreased in the SG+ and SG arms, but these changes were not significantly different from the slight increase observed in the control arm. No measurable improvements were observed for thinness.

The significant decrease in intestinal parasitic infections and anaemia could be partially explained by deworming in a 6-month interval that resulted in an increased Hb level among children in SG+. The decrease could also be explained by the number of complementary interventions to the school-aged children and their caregivers leading to increased knowledge on handwashing before eating and after defecation. Similar programmes that combined WASH and nutrition interventions in Bangladesh and Peru have shown impressive results with respect to health (increased access to safe water, improved sanitation and enhanced handwashing), reduced anaemia and improved nutritional indicators (increased DDS and reduced stunting) [24]. Our study showed no effect of the intervention on stunting and thinness and this might be explained by the fact that the increment in the duration of intervention might show an impact. Of note, height and weight may not be ideal indicators for school-aged children because of unequal growth during adolescence [25]. A study conducted in Bangladesh reported that the odds of being stunted in adolescence could be explained by the combined effect of being stunted in childhood and having mothers whose height was <145 cm [25]. Furthermore, the same study reported that girls were more likely to be stunted in childhood than boys, whereas boys were more likely than girls to be stunted in adolescence and this might be due to the difference in pace of maturation [25]. As a limitation, we did not explore the history of stunting among children in their childhood, which could be considered in future studies.

### Effects on fruits and vegetable consumption

The intervention studies conducted among children and youths have suggested that gardening can lead to improvements in fruit and vegetable consumption [5, 26, 27]. Published studies have measured the relationship between school-aged children’s fruit and vegetable intake and participation in a school garden programme. The results were, however, inconsistent for comparison with our study that only revealed a minor effect [1–3, 5, 26, 28]. Studies conducted among school-aged children reported significant beneficial effect on fruit and vegetable intake [5, 28]; one study reported a significant beneficial effect of school garden on vegetable consumption only [3]; another study reported only minor effects of school garden on fruit and vegetable intake [2]; one study found a significant beneficial effect on fruit and vegetable consumption in boys only [26]; while, yet another one study reported no differences between boys and girls in fruit and vegetable intake [1]. Christian et al. (2014) found little evidence to support that school gardens alone could improve students’ fruit and vegetable intake. The authors though reported that when the school garden programme was integrated within an educational component (curriculum), students’ daily fruit and vegetable consumption significantly increased, which is in line with the findings of our study, showing a small effect on the consumption of fruits and vegetables and growth indicators.

### Effects on the school curriculum and involvement of school-aged children and teachers for school gardening

The main aim of SG in the VgtS project was to introduce children to basic gardening skills such as land levelling, raising beds for drainage and easy planting, watering, weeding and harvesting. Only 2 weeks on every Friday, for 90 min were allocated for school garden education. Previous successful gardening interventions all involved additional elements to the gardening activities, such as health promotion programmes [1, 2, 28, 29]. In our study, we found positive impacts on children’s fruit and vegetable intake, anaemia status and intestinal parasite infections when schools integrated gardening activities throughout their curriculum and implemented additional complementary interventions (SG+). However, experiences and lessons learned are that for sustainability of the programme, schools need continued support for the provision of regular refreshment trainings on knowledge related to the gardening, health, nutrition and WASH. Of note, the successful interventions in prior trials were implemented by teachers [1–3, 28], which was only partly the case in our study.

### Effects on water quality

In our survey, some water samples from both SG and SG+ households exceeded the national tolerance limit for TTC contamination (<1 colony forming unit (CFU)/100 ml). The microbiological analyses of water samples revealed the presence of TTC in 25 water samples of SG with eight of these samples having TTC >100 CFU/100 ml; and 17 water samples of SG+ with 10 of these samples having TTC >100 CFU/100 ml that call for specific treatment. Of note, despite households reporting of obtaining water from improved sources and treating water, faecal contamination was still observed in most of the water samples. The increased water contamination with TTC might have been caused by garbage discarded in open spaces in close proximity to drinking water points, open defecation practices or cross-contamination between water supply and sewage system, leaky pipes contaminating the water via runoff or behavioural practices during transportation. Similar findings of cross-contamination and leakage points, old pipelining and drainage system and back siphoning have been reported in a study conducted in Myagdi district and a mountainous region of Nepal [30, 31].

Taken together, our study showed that combining school garden, WASH, regular deworming and nutrition interventions resulted in decreased intestinal parasitic infection and increased knowledge of children about requirement of consumption of more than five portions of fruits and vegetables per day. This might be due to addressing the immediate cause of under-nutrition (i.e. providing awareness about requirement of consumption of nutrient-dense fruits and vegetables via school garden) as well as addressing underlying contributing factors that included lack of access to clean water and sanitation, recurrent infectious diseases and lack of awareness on health and hygiene.

### Study limitations

The main issues encountered were related to difficulties in implementing SG and SG+ interventions in our study, explained by the relatively short implementation period. It is conceivable that school gardens require longer term commitment, and a supportive team for protecting and maintaining garden over the regular days as well as during school holidays. There are several limitations to our study.

First, although, the number of clusters in the intervention and control arms was the same, the numbers of children within the clusters and between the two districts were different. This is mainly explained by the challenge posed by the April 2015 earthquake, which affected particularly the Ramechhap district. Indeed, 26 children and 89 households were lost during follow-up. Approximately one out of six households (15.8%) were not found in the post-earthquake emergency crises and a number of villages were severely destroyed during the earthquake. In addition, around 3.7% of the school-aged children were lost to follow-up, due to the aftermath of the earthquake, mostly in the intervention schools, which resulted into a loss of statistical power. Second, the numbers of schools selected in Dolakha and Ramechhap districts were not equal, which might be a limiting factor in generalizing the regional differences. Third, only two of the schools had a school meal programme which, however, due to limited resources, targeted only school-aged children up to the fourth grade. Fourth, the integrated agriculture, nutrition and WASH interventions were implemented only for a relatively short period (5 months) due to delayed project implementation, a major earthquake, an economic blockage between India and Nepal and the end of the project in 2016 that might have limited the larger potential benefits for children’s health and nutritional status. Fifth, we did not explore the history of stunting among children in their childhood, which should be investigated in future studies. Sixth, we did not collect data in different seasons. Instead, the data were collected over a bit more than of a single calendar year with different fruits and vegetables being abundant in different periods of the year. This suggests that the true relationships between school gardens and nutrition outcomes, including fruit and vegetable consumption, may have been underestimated for some schools, if data were collected during the low production month. In the meantime, it is conceivable that schools, opting to maintain a vegetable garden, may be generally more interested in creating a healthier school environment [32]. Seventh, nutritional and WASH practices of children were self-reported and changes in behaviour were not closely observed, which may have resulted in over- or under-reporting. Similarly, it is conceivable that households tend to under- or over-report their dietary consumption patterns and either over- or underestimate their consumption of healthy foods, such as fruits and vegetables, thus resulting in biases of food intake assessment [33]. Eighth, the results from selected schools, households and communities in the Dolakha and Ramechhap districts may not be considered as representative for other parts of Nepal. Ninth, our diagnostic approach consisted of the collection of a single stool sample per child, which was subjected to duplicate Kato-Katz thick smear examination. The collection of multiple consecutive stool samples (instead of single specimens) and examination of triplicate or quadruplicate Kato-Katz thick smears would have resulted in higher sensitivity of the diagnostic methods [34]. Although our diagnostic approach for helminth consisted of the collection of a single stool sample per child, stool samples were subjected to multiple diagnostic methods (e.g. Kato-Katz, formalin ether-concentration and wet mount methods), which enhanced diagnostic accuracy. Tenth and finally, a limitation is that anaemia can be caused by multiple and complex factors. Thus, by using a HemoCue device for Hb measurement, the identification of the exact type of anaemia was not possible and we did not collect data on other important risk factors for anaemia, such as vitamin A, riboflavin and folate deficiencies [35].

Despite these limitations, the current research provides some evidence that SG+ interventions improve direct and indirect determinants of children’s nutritional and health indices, by reducing intestinal parasitic infections, improving Hb levels and improving certain hygiene practices. Our model of interventions implemented in these pilot schools could be readily replicated and scaled-up. The study thus holds promise to impact on public health. The methodology used for the study presents a suitable approach for evaluating impacts of school-based programme in a setting where there is paucity of information related to school-aged children’s health and nutrition. School gardens and complementary nutrition and WASH interventions could sustainably impact children’s dietary and hygiene behaviour in the longer term, if they are linked with a greater involvement of their parents/caregivers.

## Conclusions

Our study suggests that a holistic approach of school gardens, coupled with complementary education, nutrition, WASH and health interventions holds promise to increase children’s fruit and vegetable consumption and decrease intestinal parasitic infections. We recommend that engaging children into high quality gardening interventions that can also incorporate additional intervention components, such as regular deworming and educational activities (e.g. health promotion programmes and teaching children and their caregivers about healthy foods and hygiene practices) are essential for improving children’s dietary intake and health status.

## Supplementary information

**Additional file 1.** School Children Questionnaire.

**Additional file 2.** Household Questionnaire.

**Additional file 3.** Identification Codes.

**Additional file 4.** Sheet for Anthropometrics and Biomedical Specimen.

**Additional file 5: Table S1.** Changes in key indicators from questionnaire among households in two districts of Nepal, March-May 2015 and June 2016.

## Data Availability

The data analysed for this study are not publicly available, as they are part of the PhD study of the first author. However, the data are available from the corresponding author upon reasonable request and signature of a mutual agreement. The questionnaires in English are available upon request from the corresponding author.

## Abbreviations

CFU: Colony forming unit
CI: Confidence intervals
EPG: Eggs per gram of stool
Hb: Haemoglobin
KAP: Knowledge, attitude and practices
NARC: Nepal Agricultural Research Council
OR: Odds ratio
SG: School Garden
SG+: School garden with complementary intervention
TTC: Thermo-tolerant coliforms
VgtS: Vegetables go to School (project)
WASH: Water, sanitation and hygiene
WHO: World Health Organization
