# Supplementary material for: Nutritional and health status of children 15 months after integrated school garden, nutrition, and water, sanitation and hygiene interventions: a cluster-randomised controlled trial in Nepal
Source: BMC Public Health. 2020 Feb 3;20:158. doi: 10.1186/s12889-019-8027-z (PMC6998817; doi:10.1186/s12889-019-8027-z)
Supplement: Supplementary file 1 — Additional file 1. School Children Questionnaire. [file 12889_2019_8027_MOESM1_ESM.docx]

**Additional File 1: School Children Questionnaire**

Evaluation of integrated interventions in nutrition, water, sanitation, hygiene and health in the framework of the « Vegetables go to School Project » in Nepal

| 1. Questionnaire information | | |
| --- | --- | --- |
| 1.1 | Interviewer name*:* |  |
| 1.2 | Start time of the interview |  |
| 1.3 | Date of the interview |  |
| 1.4 | *Enter the ID-Code of the respondent*  Site/School  Household  Individual | \| ID-Code : \|  \|  \|  \| \| --- \| --- \| --- \| --- \| \|  \|  \|  \| \|  \| Site \| HH \| Individual \| |
| 1.5 | Has the child given his/her oral consent to be interviewed ? | □ Yes ⁭  □ No ⁭ |
| 1.6 | If the interview with the child is not possible, please note the reason |  |

| 1. Demographics | | |
| --- | --- | --- |
| 2. 1 | Sex of the respondent child | □ Male ⁭  □ Female⁭ |
| 2. 2 | What is your date of birth? | ___ /_____ /______  day/month/year |
| 2.3 | What is your age?  *(Verify with the date of birth given, if possible)* |  |
| 2.4 | Education level : Which class are you in? |  |

| 1. Nutrition | |
| --- | --- |
| **A. Nutrition: practices and habits** | |
| *Now, I would like to ask you questions with regards to the foods that you have eaten yesterday. This means, everything that you have eaten since you woke up yesterday morning until the evening and also, if you have eaten something in the night.* | |
| 3.1 | **Was yesterday a special day (for you), for example …** *(Prompt the answers, multiple answers possible)*  01 Market day 05 Other : _____________________  02 Festival day 05 Not a special day  03 Fasting day 88 Don’t know (DK)  04 Sunday 99 No Response (NR) |
| 3.2 | **Was your food intake yesterday other than normal, in terms of quantity or quality?**  01 Yes 02 No 88 DK / 99 NR |
| 3.3 | **Did you feel sick yesterday?**  01 Yes 02 No 88 DK / 99 NR |
| 3.4. | **Did you take any medication yesterday?**  01 Yes 02 No 88 DK / 99 NR |
| 3.5 | **Did you eat anything yesterday morning (breakfast)? (If NO*🡪* 3.9)**  01 Yes 02 No 88 DK / 99 NR |
| 3.6 | **If yes, at what time did you eat yesterday morning (the breakfast)?**  01 Between 6h and 7h 04 Other: _________________  02 Between 7h and 8h 88 DK  03 Between 8h and 9h 99 NR |
| 3.7 | **If yes, where did you eat yesterday morning (the breakfast)?**  01 At home 04 Other: _________________  02 In school 88 DK  03 On the road 99 NR |
| 3.8 | **If yes, what did you eat yesterday morning (for breakfast)?** *(Please not all the foods that the child ate yesterday morning)*  ______________ |
| 3.9 | **Did you eat anything yesterday at lunchtime (for lunch)? (If NO *🡪3.13)***  01 Yes 02 No 88 DK / 99 NR |
| 3.10 | **If yes, at what time did you eat lunch yesterday?**  01 Between 11h and 12h 04 Other: _________________  02 Between 12h and 13h 88 DK  03 Between 13h and 14h 99 NR |
| 3.11 | **If yes, where did you eat yesterday at lunchtime (for lunch)?**  01 At home 04 Other: _________________  02 In school 88 DK  03 On the road 99 NR |
| 3.12 | **If yes, what did you eat yesterday at lunchtime (for lunch)?** *(Please not all the foods that the child ate yesterday for lunch)*  ______________ |
| 3.13 | **Did you eat anything yesterday evening (for supper)? (If NO *🡪 3.17*)**  01 Yes 02 No 88 DK / 99 NR |
| 3.14 | **If yes, at what time did you eat supper yesterday in the evening?**  01 Between 17h and 19h 04 Other: _________________  02 Between 19h and 21h 88 DK  03 Between 21h and 24h 99 NR |
| 3.15 | **If yes, where did you eat for supper yesterday in the evening?**  01 At home 04 Other: _________________  02 Restaurant 88 DK  03 On the road 99 NR |
| 3.16 | **If yes, what did you eat for supper yesterday in the evening?** *(Please not all the foods that the child ate yesterday for supper in the evening)*  ______________ |
| 3.17 | **Did you eat anything yesterday, during the day in-between meals or in the night? (If NO *🡪 3.19*)**  01 Yes 02 No 88 DK / 99 NR |
| 3.18 | **If yes, what did in-between meals or in the night yesterday?** *(Please not all the foods that the child ate yesterday in-between meals or during the night)*  ______________ |
| **B. Nutrition: knowledge and attitudes** | |
| 3.19 | **Did you ever hear about malnutrition?**  01 Yes 02 No***🡪 3.22*** 88 DK / 99 NR ***🡪 3.22*** |
| 3.20 | **If yes, do you think that malnutrition is a problem?**  01 Yes 02 No 88 DK / 99 NR |
| 3.21 | **In your opinion, what are the possible causes of malnutrition?**  *(Do not prompt the answers, multiple answers possible)*  01 Diseases 05 Lack of means to afford good foods  02 Lack of food 06 Other : _______________________  03 Unbalanced food intake 88 DK  04 Poorly prepared food 99 NR |
| 3.22 | **How do you feel in class in the morning, if you did not eat anything before coming to school?**  00 I always eat before I go to school  01 I have difficulties to concentrate and be attentive  02 I cannot study well and have difficulties to follow the lectures  03 Other: ________________________________  88 DK 99 NR |
| 3.23 | **In your opinion, how many times should you eat vegetable and fruits per day (i.e. a portion, piece, whole fruit, etc.)**  01 5 per day 06 None per day  02 4 per day 07 More than 5 per day  03 3 per day 08 Other : ________________________  04 2 per day 88 DK  05 1 per day 99 NR |
| 3.24 | **In your opinion, is it good or not good for your health to eat a variety of vegetables and fruits?**  01 It is not good 04 Other : ________________________  02 I am not sure 88 DK  03 It is good 99 NR |
| **4. Water, sanitation and hygiene (WASH)** | |
| **A. WASH: practices and habits** | |
| 4.1 | **On what occasion(s) do you wash your hands with soap (detergent or similar)?** *(Prompt the answers!)*  01 Before eating □ Yes □ No  02 After eating □ Yes □ No  03 After playing (something) □ Yes □ No  04 After the toilet □ Yes □ No  05 Other : ________________ □ Yes □ No  06 I don’t wash my hands with soap  88 DK 99 NR |
| 4.2 | **Why are you washing your hands with soap?**  ________________ |
| 4.3 | **What are you usually using when washing your hands?** *(Do not prompt the answers, multiple answers possible)*  01 Water 04 Soap  02 Ash 05 Other : ________________________  03 Mud, soil 88 DK  99 NR |
| 4.4 | **At what occasion(s) should you wash your hands with soap?** *(Prompt the answers!)*  01 Before eating □ Yes □ No  02 After eating □ Yes □ No  03 After playing (something) □ Yes □ No  04 After the toilet □ Yes □ No  05 Other : ________________ □ Yes □ No  88 DK 99 NR |
| 4.5 | **Do your brush your teeth?**  01 Yes 04 Other : ________________________  02 No ***🡪 4.7*** 88 DK  03 Sometimes 99 NR |
| 4.6 | **With what frequency do you brush your teeth?**  01 Never 05 Once every two weeks  02 Every day 06 Once per month  03 Every second day 07 Other : ________________________  04 Once per week 08 Multiple times per day  99 NR 88 DK |
| 4.7 | **Do you drink the water that is provided at your school?**  01 Yes 05 Other : ________________________  02 No 88 DK  03 Sometimes 99 NR  04 No water is provided at the school |
| 4.8 | **If you come to school, are you bringing water from home to drink?**  01 Yes 04 Other : ________________________  02 No 88 DK  03 Sometimes 99 NR |
| 4.9 | **Do you sometimes … in a river, swamp or lake?** *(Prompt the answers!)*  **… play** □ Yes □ No □ DK □ NR  **… fish?** □ Yes □ No □ DK □ NR  **… wash ?** □ Yes □ No □ DK □ NR  **… work ?** □ Yes □ No □ DK □ NR |
| 4.10 | **Are there any latrines at your school?**  01 Yes 02 No ***🡪 4.13*** 88 DK***🡪 4.13*** 99 NR |
| 4.11 | **Do you use the latrines at your school?**  01 Yes ***🡪 4.14*** 02 No 88 DK 99 NR |
| 4.12 | **Why do you not use the latrines at your school?**  01 Dirty 06 Not enough latrines  02 No water 07 No intimacy  03 No soap 08 Other : ________________________  04 Bad odour, smell 88 DK  05 Not functional 99 NR |
| 4.13 | **Where do you defecate instead, if you are in class but not using the latrines at school?**  01 Bush 04 Other : ________________________  02 Behind the latrines 88 DK  03 At home 99 NR |
| **B. WASH: knowledge and attitudes** | |
| 4.14 | **In your opinion, can dirty water cause diseases/illness?**  01 Yes 02 No ***🡪 4.16*** 88 DK 99 NR |
| 4.15 | **What kind of diseases/illnesses can be caused by dirty water?** *(Do not prompt the answers, multiple answers possible)*  01 Diarrhea 06 Malaria  02 Cholera 07 Eye irritations, diseases  03 Skin irritations 08 Schistosomiasis  04 Icterus 09 Worms, parasites  05 Typhus 10 Other : ________________________  88 DK 99 NR |
| 4.16 | **Do you think that if you do not wash your hands, there is a risk that you can become sick, for example having stomach ache or diarrhea?**  01 Yes 04 Other : ________________________  02 No 88 DK  03 Not sure 99 NR |
| 4.17 | **Do you think that if you do not brush your teeth thee is a risk that you can have tooth pain?**  01 Yes 04 Other : ________________________  02 No 88 DK  03 Not sure 99 NR |

| **End of Questionnaire** | | |
| --- | --- | --- |
| 5.1 | Do you have any questions? |  |
| 5.2 | Remarks from the interviewer: |  |
| 5.3 | End time of the interview: |  |
| **We are now finished with the interview, thank you very much for your participation!** | | |
| 5.4 | Remarks from the supervisor: |  |
