# Supplementary material for: Nutritional and health status of children 15 months after integrated school garden, nutrition, and water, sanitation and hygiene interventions: a cluster-randomised controlled trial in Nepal
Source: BMC Public Health. 2020 Feb 3;20:158. doi: 10.1186/s12889-019-8027-z (PMC6998817; doi:10.1186/s12889-019-8027-z)
Supplement: Supplementary file 2 — Additional file 2. Household Questionnaire. [file 12889_2019_8027_MOESM2_ESM.docx]

**Additional File 2: Evaluation of integrated nutrition, water, sanitation and hygiene interventions in the framework of the « Vegetables go to School – Project » in Nepal:**

**Household Questionnaire**

|  | Name | Signature |
| --- | --- | --- |
| Parent, caretaker, household head |  |  |
| Selected child(ren) |  | Oral consent : □ Yes □ No |
| Interviewer |  |  |

| ID-Code:  (parent) |  |  |  |  | ID-Code:  (child) |  |  |  |  |  |
| --- | --- | --- | --- | --- | --- | --- | --- | --- | --- | --- |
|  |  |  |  |  |  |  |  |  |  |  |
|  | Site | Grade | Individual |  |  | Site | Grade | Child |  | Date (Day/Month/Year) |

| VgtS Project : Household Questionnaire |
| --- |
| \| 0.1 Interviewer name: __________________________ \| \| --- \| \| 0.2 Start time of the interview __________________________ \| \| 0.3 Interview date __________________________ \| \| 0.4 ID-Code of respondent __________________________ \| \| 0.5 GPS coordinates of the household __________________________ \| \| 0.6 Number of children selected in the HH ______ \| \| 0.7 ID-Code(s) of child(ren) : _______________ _________________ \| \| 0.8 Has the respondent given his consent? □ Yes □ No \| |

***Interviewers: Please conduct the household questionnaire with the mother of the selected child or the person in charge (caregiver).***

| 1. Demographics and socioeconomic data | | | | |
| --- | --- | --- | --- | --- |
| 1.1 | **What is your date of birth?**  _______ / ________ / ____________ 88 DK | | | |
| 1.2 | **What is your age?** Age _____ (years) 88. DK 99. NR | | | |
| 1.3 | **What is your education level?**  01 No education 05 Secondary complete  02 Primary incomplete 06 Superior  03 Primary complete 07 Other | | | |
| 1.4 | **What is the principal occupation of the household head?**  01 Farmer 05 Labour  02 Public services 06 No occupation  03 Small business owner 07 Other:  04 Large business owner 08 NR | | | |
| 1.5 | ***Observation*:** *Principal material of the roof*  01 No roof 03 Tiles  02 Grass 04 Corrugated iron roof  03 Wood 05 Other: | | | |
| 1.6 | ***Observation*:** *Principal material of the walls*  01 Grass 03 Bricks  02 Wood 04 Corrugated iron  03 Mud 05 Other: | | | |
| 1.7 | ***Observation*:** *Principal material of the soil*  01 Soil, sand 05 Carpet  02 Mud 06 Bricks, burnt  03 Cement 07 Bricks, dried  04 Wood 08 Other: | | | |
| 1.8 | **What type of fuel does your household principally use to cook?**  01 Electricity 05 Other:  02 Gas, petrol 06 HH does not cook  03 Coal 07 DK  04 Wood 08 NR | | | |
| 1.9 | **Where do you usually do the cooking? *Observation!***  01 Indoors, in the main building 04 Outdoors  02 Indoors, in a separate building 05 Other:  03 In another building 06 NR | | | |
| 1.10 | **What energy source(s) does your household use?** *Multiple answers possible!*  00 No energy source 06 Private grid  01 Gas, petrol 07 Generator  02 Coal 08 Other :  03 Wood 09 DK  04 National (public) grid 10 NR  05 Solar panel | | | |
| 1.11 **In your household, do you or anyone possess …?**   1. A radio? 01. Yes 02. No 88. DK 99. NR 2. A television? 01. Yes 02. No 88. DK 99. NR 3. A mobile phone? 01. Yes 02. No 88. DK 99. NR 4. A table? 01. Yes 02. No 88. DK 99. NR 5. A stove? 01. Yes 02. No 88. DK 99. NR 6. A petrol lamp? 01. Yes 02. No 88. DK 99. NR 7. A gas lamp? 01. Yes 02. No 88. DK 99. NR 8. A cupboard? 01. Yes 02. No 88. DK 99. NR 9. An iron? 01. Yes 02. No 88. DK 99. NR 10. A watch? 01. Yes 02. No 88. DK 99. NR 11. A bike? 01. Yes 02. No 88. DK 99. NR 12. A motorbike? 01. Yes 02. No 88. DK 99. NR 13. A car or truck? 01. Yes 02. No 88. DK 99. NR | | | | |
| 1.12 | **Does your household own or use any agricultural land?**  01 Yes 02 No 99 NR | | | |
| 1.13 | **Of the total production of your agricultural land, what percentage do you think are you using for your household’s consumption?**  01. ≤ 10 02. 10-13 % 03. 30-50% 04. 50-70% 05. >70% 88. DK 99. NR | | | |
| 1. Water, sanitation and hygiene | | | | |
| 2.1 | **What is the households’ principal source of drinking water during the dry season?** *(do not prompt the answers)*  01 Private tap (in the court) 07 Surface water (river, lake, swamp, ponds, …)  02 Shared tap (with neighbours) 08 Deep-well, bore hole  03 Public tap 09 Other:  04 Improved source 88 DK  05 Non-improved source 99 NR  06 Hand-pump | | | |
| 2.2 | **What is the households’ principal source of drinking water during the rainy season?** *(do not prompt the answers)*  01 Private tap (in the court) 07 Surface water (river, lake, swamp, ponds, …)  02 Shared tap (with neighbours) 08 Deep-well, bore hole  03 Public tap 09 Other:  04 Improved source 88 DK  05 Non-improved source 99 NR  06 Hand-pump 99 NR | | | |
| 2.3 | **How many minutes does it take from your household to the principal source and back to fetch drinking water?**  01. ≤ 15’ 02. 15’-30’ 03. 30’-60’ 04. > 60’ 88. DK 99. NR | | | |
| 2.4 | **What kind of container do you usually use to fetch drinking water at the principal source?**  01 Clay pot 06 local  02 Plastic container 07 local  03 Metal container 08 Other:  04 local 88 DK ***🡪 2.10***  05 local 99 NR ***🡪 2.10*** | | | |
| 2.5 | **What kind of container do you usually use to store the drinking water in your household before drinking?**  01 No drinking water storage 06 local  02 Clay pot 07 local  03 Plastic container 08 Other:  04 Metal container 88 DK ***🡪 2.10***  05 local 99 NR ***🡪 2.10*** | | | |
| 2.6 | ***Observation:*** *Is the container covered during storage?*  01 Covered 02 Uncovered 03 Not seen 99 NR | | | |
| 2.7 | **At what frequency is the container washed with soap?**  00 Never 03 Other:  01 _____ per day 88 DK  02 _____ per week 99 NR | | | |
| 2.8 | **Is the same container used for any other activity?**  01 Yes 02 No ***🡪 2.10*** 88 DK ***🡪 2.10*** 99 NR ***🡪 2.10*** | | | |
| 2.9 | **If yes, what is it used for?**  _____________________ | | | |
| 2.10 | **Are you treating your drinking water in any way to make it safer to drink?** *(Do not prompt answers – multiple answers possible)*  01 No treatment 08 Sand filtration  02 Boil 09 local  03 Solar disinfection 10 local water treatment product name  04 Add chlorine / javel 11 Other:  05 Let it rest and settle (sedimentation) 88 DK  06 Filtration with a tissue 99 NR  07 Filtration with a ceramic filter | | | |
| 2.11 | **In your opinion, what are the risks of drinking unclean water?** *(Do not prompt answers – multiple answers possible)*  01 Diarrhea 06 Polio  02 Malnutrition 07 Other:  03 Stomach problems / aches 88 DK  04 Bad digestion 99 NR  05 Worms | | | |
| 2.12 | **Does your household have a (own) functional latrine/WC/toilet?**  01 Yes ***🡪 2.14*** 02 No | | | |
| 2.13 | **If not, where do you defecate?**  01 Shared latrine 03 River, swamp, lake, etc.  02 Bush 04 Other :  99 NR | | | |
| 2.14 | **Please show me the place of your latrine/WC/toilet. *Observation!*** *Observe and note the type of latrine/ toilette:*  01 VIP latrine 05 Other  02 Open pit latrine with slab 06 Don’t know  03 Open pit latrine without slab 99 NR  04 Flushed toilet | | | |
| 2.15 | **Does your household have soap/detergent which can be used for washing hands?**  01 Yes, soap seen 88 DK  02 Yes, but soap not seen 99 NR  03 No | | | |
| 2.16 | **What are you usually using when washing your hands?**  01 Water only 05 Water and soap  02 Ash only 06 Water and ash  03 Sand only 07 Water and sand  04 Mud only 08 Other:  99 NR | | | |
| 2.17 | **At what occasion(s) are you washing your hands with soap/detergent/disinfectant?** *(Prompt answers!)*  01 Before preparing food □ Yes □ No  02 Before eating □ Yes □ No  03 After eating □ Yes □ No  04 After the toilet □ Yes □ No  05 After the child’s defecation □ Yes □ No  06 Before breastfeeding □ Yes □ No  07 After breastfeeding □ Yes □ No  08 Other : ________________ □ Yes □ No  09 I don’t wash my hands with soap □ Yes □ No  88 DK  99 NR | | | |
| 2.18 | **How is your household deposing of the solid waste?**  01 Throw in the court or backyard 05 Throw on fields  02 Burn 06 Is collected at household and disposed  03 Throw into a pit 07 Other:  04 Burry 88 DK  99 NR | | | |
| 2.19 | **Does your household own any livestock?**  00 No livestock 05 Donkey(s)  01 Poultry 06 Cow(s)  02 Cat(s) 07 Swine  03 Dog(s) 07 Other:  04 Goat(s) 08 NR | | | |
| 2.20 | **Please show me the place where you keep your animals/livestock? *Observation!***  01 Livestock/animals are free in the court 04 Other:  02 Livestock/animals are fenced in the court 05 Not observed  03 Livestock/animals are outside the court 99 NR | | | |
| 2.21 | **Do you ever have animals inside of your household (within the building)?** *(Also observation!)*  01 Yes 02 No 88 DK 99 NR | | | |
| 1. Child health | | | | |
| 3.1 | **What is the ethic group of the selected child(ren)?**  01 05  02 06  03 07 Other:  04 99 NR | | | |
| 3.2 | **What is the religion of the selected child (ren)?** *(Multiple answers possible!)*  01 05  02 06  03 07 Other:  04 99 NR | | | |
| 3.3 | **How many children of your household were selected to participate in the survey?**  01 One child 02 Two children 03 Three children | | | |
| 3.4 | ***Note the ID-Code of the selected child(ren)!***  *(If more than one children were selected, ask the following section for every single child!)* | | | |
|  | \| ID-Code : \|  \|  \|  \| \| --- \| --- \| --- \| --- \| \|  \|  \|  \| \|  \| Site \| HH \| Child 1 \| | | \| ID-Code : \|  \|  \|  \| \| --- \| --- \| --- \| --- \| \|  \|  \|  \| \|  \| Site \| HH \| Child 2 \| | |
|  | \| ID-Code : \|  \|  \|  \| \| --- \| --- \| --- \| --- \| \|  \|  \|  \| \|  \| Site \| HH \| Child 3 \| | |  | |
| 3.6 | **Has {NAME} suffered from … in the past 14 days / past 2 weeks?** (*If all responses are No 🡪 3.7_PM)* | | | |
| 1. Fever 01 Yes 02 No 88 DK 99 NR 2. Cough 01 Yes 02 No 88 DK 99 NR 3. Respiratory difficulties /   Increased respiratory rhythm 01 Yes 02 No 88 DK 99 NR   1. Diarrhea 01 Yes 02 No 88 DK 99 NR 2. Blood in stool 01 Yes 02 No 88 DK 99 NR 3. Mucus in stool 01 Yes 02 No 88 DK 99 NR 4. Blood in urine 01 Yes 02 No 88 DK 99 NR | | | | |
| 3.7 | **Did you seek medical advice or treatment for the condition/illness?** *(Any of the conditions/illnesses mentioned in 3.6)*  01 Yes 02 No **🡪 *3.9*** 88 DK 99. NR | | | |
| 3.7_PM | **If your child(ren) is/are sick, where do you normally seek medical advice or treatment?** *(Do not prompt answers – multiple answers possible) – Jump to* ***🡪 3.10*** *after this question!*  01 Hospital 06 Traditional medicine  02 Health centre 07 Pharmacy  03 Health post 08 Other:  04 Community health worker 88 DK  05 Self-treatment 99 NR | | | |
| 3.8 | **Where did you seek medical advice or treatment?** *(Do not prompt answers – multiple answers possible) – Jump to* ***🡪 3.10*** *after this question!*  01 Hospital 06 Traditional medicine  02 Health centre 07 Pharmacy  03 Health post 08 Other:  04 Community health worker 88 DK  05 Self-treatment 99 NR | | | |
| 3.9 | **Why did you not seek medical advice or treatment?** *(Do not prompt answers – multiple answers possible)*  01 Accessibility: I do not have good access to a health facility  02 Acceptability: I do not like services/personnel/structures at the health facility  03 Affordability: I do not have money to go to the health facility  04 I prefer to go to the traditional/folk healer and/or treat myself  05 It was not necessary to go to the health facility  06 I did not have the permission to go to the health facility  07 I was afraid that there would be no female provider  08 Other: | | | |
| 3.10 | **If a child has diarrhea, what should you do?** *(Do not prompt answers – multiple answers possible)*  01 Give ORS  02 Give less food than usual  03 Give the same amount of food as usual  04 Give more food than usual  05 Give less liquids than usual  06 Give the same amount of liquids as usual  07 Give more liquids than usual  08 Give syrup  09 Give traditional medicine, remedies, herbs, etc.  10 Give treated water  11 Give juice of … (local)  12 Seek advice and treatment at a health centre  13 Other:  88 DK  99 NR | | | |
| 3.11 | **Have you ever heard of ‘intestinal parasites’?**  01 Yes 02 No ***🡪 4.1*** | | | |
| 3.12 | **What can be done to protect children against intestinal parasites?** *(Do not prompt answers – multiple answers possible)*  01 Wash hands with soap  02 Cut finger nails  03 Wear pants, trousers  04 Wash fruit and vegetables before consumption  05 Wear shoes  06 Drink clean water  07 Take medication  08 Other:  88 DK  99 NR | | | |
| 1. Nutrition | | | | |
| 4.1 | **Have you ever heard of malnutrition?**  01 Yes 02 No ***🡪 5.2*** 99 NR ***🡪 4.3*** | | |  |
| 4.2 | **What are the causes of malnutrition?** *(Do not prompt answers – multiple answers possible)*  01 Food quantity is insufficient / lack of food  02 Irregular meals/food consumption  03 Diseases  04 Early weaning  05 Negligence when breastfeeding  06 Lack of food diversity  07 Other:  88 DK  99 NR | | |  |
| 4.3 | **Did you breastfeed {NAME}?** *(If multiple children selected in the household, ask about the youngest)*  01 Yes 02 No ***🡪 4.5*** | | |  |
| 4.4 | **When did you start to breastfeed this child after you gave birth to him/her**?  01 Immediately 02 Hours: _ _ 03 Days: _ _ 88. DK 99 NR | | |  |
| 4.5 | **What else did you give?** *(Do not prompt answers – multiple answers possible)*  01 Nothing ***🡪 4.7*** 06 Glucose  02 Ordinary water 07 Herbs  03 ORS / Jeevan Jal 08 Rice pap  04 Rice water 09 Others: _ _ _ _ _ _ _ _ _ _ _ _  05 Milk powdered | | |  |
| 4.6 | **When did you start with these?**  01 Immediately after birth 03 After the second vaccination  02 After the first vaccination 04 After the third vaccination  88 DK 99 NR | | |  |
| 4.7 | **Have you ever heard of night blindness?**  01 Yes. 02 No 99 NR | | |  |
| 4.8 | **Have you ever heard of Vitamin A?**  01 Yes 02 No ***🡪 4.12*** 99 NR ***🡪 4.12*** | | |  |
| 4.9 | **Why is Vitamin A important for us?** *(Do not prompt answers – multiple answers possible)*  01 It is good for our health 04 Other:  02 It is protecting us against diseases 88 DK  03 It is good for the eyes / vision 99 NR | | |  |
| 4.10 | **Can you name examples of Vitamin A-rich foodstuffs?** *(Do not prompt answers – multiple answers possible)*  01 Organ meat 06 Palm oil (red)  02 Eggs 07 Foods that were enriched with Vit A  03 Diary products 08 Fish  04 Dark green vegetables 09 Other:  05 Orange fruits and vegetables 88 DK  99 NR | | |  |
| 4.11 | **How can you prevent a Vitamin A deficiency?** *(Do not prompt answers – multiple answers possible)*  01 Eat Vitamin A-rich foodstuffs  02 Eat foodstuffs that were enriched with Vitamin A  03 Give Vitamin A supplements  04 Other: 88 DK 99 NR | | |  |
| 4.12 | **Have you ever heard of anaemia?**  01 Yes 02 No ***🡪 4.14*** 99 NR ***🡪 4.14*** | | |  |
| 4.13 | **What are the causes of anaemia?** *(Do not prompt answers – multiple answers possible)*  01 Lack of iron in the consumed foodstuffs  02 Lack of enough foodstuffs  03 Diseases and infections (malaria, intestinal parasites, HIV/AIDS, etc.)  04 Heavy bleeding during menstruation  05 Other:  06 Other:  88 DK  99 NR | | |  |
| 4.14 | **Why is iron important for children?**  01 Against deficient mental development  02 Against deficient physical development  03 Other:  88 DK  99 NR | | |  |
| 4.15 | **Can you name examples of iron-rich foodstuffs?** *(Do not prompt answers – multiple answers possible)*  01 Meat 04 Iron-enriched foodstuffs  02 Fish and seafood 05 Other :  03 Dark green vegetables 88 DK  99 NR | | |  |
| 4.16 | **How many times did your child eat at the school canteen this week?**  00 0 times / never 05 5 times  01 1 times 06 6 times  02 2 times 07 There is no canteen at the school  03 3 times 88 DK  04 4 times 99 NR | | |  |
| 4.17 | **Usually, how many meals does your child eat per day?** _____________ times | | |  |
| 4.18 | **Do you give your child other things to eat, for example sweets or snacks, aside the usual meals?**  01 Yes 02 No ***🡪 4.19*** | | |  |
| 4.18_A | **If yes, what do you give?**  ____________________________________________________ | | |  |
| 4.19 | **Do you give your child any food supplements, for example sarbottam pitho aside the usual meals?**  01 Yes 02 No ***🡪 4.20*** | | |  |
| 4.19_A | **If yes, what supplements do you give?** ____________________________________________________ | | |  |
| 4.20 | **In your opinion, are the meals that you give your child appropriate?**  01 Yes 02 No ***🡪4.21*** 88 DK 99 NR | | |  |
| 4.21 | **If not, what should be improved?**  01 Increase the quantity 04 Give a particular foodstuff: _____________  02 More diverse foodstuffs 05 Other :  03 Increase the frequency of meals 88 DK  99 NR | | |  |
| 4.22 | **Do you give/prepare your child vegetables to eat?**  01 Yes 02 No ***🡪4.26*** 88 DK***🡪4.26*** 99 NR***🡪4.26*** | | |  |
| 4.23 | **If yes, please name the vegetables that you give/prepare your child to eat:** *(Multiple answers possible)*  01 Beans 07 Salads  02 Tomatoes 08 Carrots  03 Onions 09  04 Corn 10 Other:  05 88 DK  06 99 NR | | |  |
| 4.24 | **Why are you giving/preparing your child vegetables to eat?** *(Multiple answers possible)*  01 Health personnel told me to 05 We eat what is available  02 Education at the school, teachers told me to 06 Other:  03 It is good for my child 88 DK  04 Everybody does it 99 NR | | |  |
| 4.25 | **Which factors are you considering when you choose to give/prepare your child vegetables to eat?** *(Multiple answers possible)*  01 Price, affordability 06 Texture  02 Availability 07 Seasonality  03 Freshness 08 Other:  04 Taste 88 DK  05 Colour 99 NR | | |  |
| 4.26 | **If not, why are you not giving/preparing your child vegetables to eat?** *(Multiple answers possible)*  01 Child does not like vegetables 05 Vegetables are not available  02 Child is too young to eat vegetables 06 Other  03 Texture is not good for the children 88 DK  04 Vegetables are expensive 99 NR | | |  |
| 4.27 | **Do you give your child fruits to eat?**  01 Yes 02 No ***🡪4.29*** 88 DK ***🡪4.29*** 99 NR ***🡪4.29*** | | |  |
| 4.28 | **If yes, please name the fruits that you give your child to eat:** *(Multiple answers possible)*  01 Apple 07 Guava  02 Orange 08 Banana  03 Mango 09 Pomegranate  04 Litchi 10 Other:  05 Pineapple 88 DK  06 Jackfruit 99 NR | | |  |
| 4.29 | **Why are you giving your child fruits to eat?** *(Multiple answers possible)*  01 Health personnel told me to 05 We eat what is available  02 Education at the school, teachers told me to 06 Other:  03 It is good for my child 88 DK  04 Everybody does it 99 NR | | |  |
| 4.30 | **Which factors are you considering when you choose to give your child fruits to eat?** *(Multiple answers possible)*  01 Price, affordability 06 Texture  02 Availability 07 Seasonality  03 Freshness 08 Other:  04 Taste 88 DK  05 Colour 99 NR | | |  |
| 4.31 | **If not, why are you not giving/preparing your child fruits to eat?** *(Multiple answers possible)*  01 Child does not like fruits 06 Children look themselves for fruits  02 Child is too young to eat fruits 07 Other  03 Texture is not good for the children 88 DK  04 Fruits are expensive 99 NR  05 Fruits are not available | | |  |
| 1. Food security | | | | |
| 5.1 | **How many meals per day are served/prepared in your household? How many times per day does your household eat?** ___ times | | | |
| 5.2 | **In your opinion, how many months per year has your household enough to eat?**  ____ months 88 DK 99 NR | | | |
| 5.3 | **In the past month, have you ever been worried that your household does not have enough food to eat?**  01 Yes 99. NR***🡪 5.5***  02 No 🡪 ***5.5*** | | | |
| 5.4 | **How many times was this the case in the past month?**  01 Rarely (one or two times in the past month) 88 DK  02 Sometimes (three to ten times in the past month) 99 NR  03 Often (more than ten times in the past month) | | | |
| 5.5 | **In the past month, have you or any member of your household eaten a limited variety of foodstuffs because you of lack of means to buy foodstuffs?**  01 Yes 99. NR***🡪 5.7***  02 No 🡪 ***5.7*** | | | |
| 5.6 | **How many times was this case in the past month?**  01 Rarely (one or two times in the past month) 88 DK  02 Sometimes (three to ten times in the past month) 99 NR  03 Often (more than ten times in the past month) | | | |
| 5.7 | **In the past month, has it occurred that your household was completely out of foodstuffs because of lack of means to buy foodstuffs?**  01 Yes 99. NR***🡪 6.1***  02 No 🡪 ***6.1*** | | | |
| 5.8 | **How many times was this the case in the past month?**  01 Rarely (one or two times in the past month) 88 DK  02 Sometimes (three to ten times in the past month) 99 NR  03 Often (more than ten times in the past month) | | | |
| 1. Water quality assessment | | | | |
| 6.1 | | **Can you please pour me a sample of your drinking water – as you give it to your children – in my sample bottle/recipient?**  *The mother should pour the household drinking water from her drinking container into the sample container. Attention: Make sure that her container and your/her hands DO NOT TOUCH the sample bottle/recipient!!* | | |
| 6.2 | | ID-Code on the bottle corresponds with the ID-Code of the HH:   \| ID-Code : \|  \|  \|  \| \| --- \| --- \| --- \| --- \| \|  \|  \| N/A \| \|  \| Site \| HH \|  \| | | |
| 6.3 | | **The water you just gave me, did you treat it in any way?**  01 No treatment 08 Sand filtration  02 Boil 09 local  03 Solar disinfection 10 local water treatment product name  04 Add chlorine / javel 11 Other:  05 Let it rest and settle (sedimentation) 88 DK  06 Filtration with a tissue 99 NR  07 Filtration with a ceramic filter | | |

**Interviewers:** *Express your thankfulness to the household members and respondent for the participation in the survey and the time sacrificed!*

Observations by the interviewer: _______________________________________________________

_________________________________________________________________________________

Observations by the supervisor:

__________________________________________________________________________________________________________________________________________________________________

End time of the interview: _____________
