# Supplementary material for: Nutritional and health status of children 15 months after integrated school garden, nutrition, and water, sanitation and hygiene interventions: a cluster-randomised controlled trial in Nepal
Source: BMC Public Health. 2020 Feb 3;20:158. doi: 10.1186/s12889-019-8027-z (PMC6998817; doi:10.1186/s12889-019-8027-z)
Supplement: Supplementary file 3 — Additional file 3. Identification Codes. [file 12889_2019_8027_MOESM3_ESM.docx]

**Additional file 3: Identification Codes**

**Household (HH) composition:**

| **ID** | **HH head** | **Position** | **Name, Family Name** | **Remarks (Tel. contact, location of HH, etc)** |
| --- | --- | --- | --- | --- |
| 01 | □ | Child selected at school (1) |  |  |
| 02 | □ | Child selected at school (2) |  |  |
| 03 | □ | Child selected at school (3) |  |  |
| 04 | □ | Mother of the child |  |  |
| 05 | □ | Father of the child |  |  |
| 06 | □ | [Other person] |  |  |
| 07 | □ | [Other person] |  |  |

| **ID-Code for household questionnaire respondent:**   \| Code-ID : \|  \|  \|  \| \| --- \| --- \| --- \| --- \| \|  \|  \|  \| \|  \| Site \| Household \| Individual / adult \|  \| Code-ID : \|  \|  \|  \| \| --- \| --- \| --- \| --- \| \|  \|  \|  \| \|  \| Site \| Household \| Individual / adult \|  \| Code-ID : \|  \|  \|  \| \| --- \| --- \| --- \| --- \| \|  \|  \|  \| \|  \| Site \| Household \| Individual / adult \| | **ID-Code for the child questionnaire respondent(s):**   \| Code-ID : \|  \|  \|  \| \| --- \| --- \| --- \| --- \| \|  \|  \|  \| \|  \| Site \| Household \| Individual / child \|  \| Code-ID : \|  \|  \|  \| \| --- \| --- \| --- \| --- \| \|  \|  \|  \| \|  \| Site \| Household \| Individual / child \|  \| Code-ID : \|  \|  \|  \| \| --- \| --- \| --- \| --- \| \|  \|  \|  \| \|  \| Site \| Household \| Individual / child \| | Sites : |
| --- | --- | --- | --- | --- | --- | --- | --- | --- | --- | --- | --- | --- | --- | --- | --- | --- | --- | --- | --- | --- | --- | --- | --- | --- | --- | --- | --- | --- | --- | --- | --- | --- | --- | --- | --- | --- | --- | --- | --- | --- | --- | --- | --- | --- | --- | --- | --- | --- | --- | --- | --- | --- | --- | --- | --- | --- | --- | --- | --- | --- | --- | --- | --- | --- | --- | --- | --- | --- |
|  |  | **🡪 These ID-Codes are to be used in the questionnaires (tablets)**  **🡪 These ID-Codes are to be used on the Informed Consent Sheet**  **🡪 These ID-Codes are to be used on the Anthropometrics and Biomedical Specimen Sheet**  **🡪 These ID-Codes are to be used on the stool, and water samples** |

□ The survey was not possible in this household. Please specify the reasons:

________________________________________________________________________
