# Supplementary material for: Nutritional and health status of children 15 months after integrated school garden, nutrition, and water, sanitation and hygiene interventions: a cluster-randomised controlled trial in Nepal
Source: BMC Public Health. 2020 Feb 3;20:158. doi: 10.1186/s12889-019-8027-z (PMC6998817; doi:10.1186/s12889-019-8027-z)
Supplement: Supplementary file 4 — Additional file 4. Sheet for Anthropometrics and Biomedical Specimen. [file 12889_2019_8027_MOESM4_ESM.docx]

**Additional file 4: Sheet for Anthropometrics and Biomedical Specimen**

| **ID-Code child:**   \| ID-Code : \|  \|  \|  \| \| --- \| --- \| --- \| --- \| \|  \|  \|  \| \|  \| Site \| Household \| Child \| |
| --- | --- | --- | --- | --- | --- | --- | --- | --- | --- | --- | --- |

|  |  | **Results** |
| --- | --- | --- |
| **Sex** |  | □ Male ⁭ □ Female ⁭ |
| **Date of birth** |  | / / |
| **Weight** |  | kg |
| **Height** |  | cm |
| **Blood sample taken** | □ Yes ⁭ □ No/Refused | Hb: g/dl |
| **Stool sample day 1 taken** | □ Yes ⁭ □ No/Refused |  |
