# Supplementary material for: Nutritional and health status of children 15 months after integrated school garden, nutrition, and water, sanitation and hygiene interventions: a cluster-randomised controlled trial in Nepal
Source: BMC Public Health. 2020 Feb 3;20:158. doi: 10.1186/s12889-019-8027-z (PMC6998817; doi:10.1186/s12889-019-8027-z)
Supplement: Supplementary file 5 — Additional file 5: Table S1. Changes in key indicators from questionnaire among households in two districts of Nepal, March-May 2015 and June 2016. [file 12889_2019_8027_MOESM5_ESM.docx]

| Supplementary Table 1: Changes in key indicators from questionnaire among households in two districts of Nepal, March/May 2015 and June 2016 | | | | | | |
| --- | --- | --- | --- | --- | --- | --- |
| **Predictors** | **Group** | **Baseline (%)** | **Endline (%)** | **Change in prevalence (%)** | **Odds ratio*** | ***p*-value** |
| WASH |  |  |  |  |  |  |
| Water sufficiency | Control | 73.4 | 78.0 | 4.6 | 1.00 |  |
|  | SG-Intervention | 83.8 | 98.2 | 14.4 | 14.6 (2.43-88.0) | **0.003** |
|  | Combined Intervention (SG+) | 82.6 | 90.9 | 8.3 | 2.03 (0.60-6.88) | 0.25 |
| Water treatment | Control | 21.2 | 71.8 | 50.6 | 1.00 |  |
|  | SG-Intervention | 9.9 | 65.8 | 55.9 | 0.89 (0.16-4.87) | 0.89 |
|  | Combined Intervention (SG+) | 0.1 | 41.3 | 41.2 | 0.20 (0.04-1.14) | 0.07 |
| Possession of own latrine | Control | 66.4 | 82.1 | 15.7 | 1.00 |  |
|  | SG-Intervention | 69.4 | 96.4 | 27.0 | 5.66 (0.86-37.4) | 0.07 |
|  | Combined Intervention (SG+) | 85.1 | 92.6 | 7.5 | 3.65 (0.64-20.8) | 0.15 |
| Soap for handwashing in household | Control | 71.8 | 78.8 | 7.0 | 1.00 |  |
|  | SG-Intervention | 78.4 | 91.0 | 12.6 | 5.22 (0.90-30.1) | 0.07 |
|  | Combined Intervention (SG+) | 80.2 | 87.6 | 7.4 | 1.39 (0.32-6.13) | 0.66 |
| SG: School garden |  |  |  |  |  |  |
| SG+: School garden, nutrition, and water, sanitation and hygiene (WASH) | | | | | | |
| *Odds ratios were obtained from mixed logistic regression models for the respective end-line outcome including the factor group and random intercepts for the schools, while also adjusting for the outcome observed at baseline, the district, sex and age of the child, and education level and socioeconomic status of caregivers. | | | | | | |
| *Chlorination water treatment was introduced in all three arms during the study period from different humanitarian organisations as well in the households, thus no statistical comparisons were made. | | | | | | |
